# Predicting Ultra-High-Performance Concrete Compressive Strength Using Tabular Generative Adversarial Networks

**DOI:** 10.3390/ma13214757

**Published:** 2020-10-24

**Authors:** Afshin Marani, Armin Jamali, Moncef L. Nehdi

**Affiliations:** 1 Department of Civil and Environmental Engineering, Western University, London, ON N6A 5B9, Canada; amarani@uwo.ca; 2 Department of Civil Engineering, K. N. Toosi University of Technology, Tehran 1969764499, Iran; armin.jamali69@gmail.com

**Keywords:** ultra-high-performance concrete, compressive strength, machine learning, tabular generative adversarial networks, random forest, extra trees, gradient boosting

## Abstract

There have been abundant experimental studies exploring ultra-high-performance concrete (UHPC) in recent years. However, the relationships between the engineering properties of UHPC and its mixture composition are highly nonlinear and difficult to delineate using traditional statistical methods. There is a need for robust and advanced methods that can streamline the diverse pertinent experimental data available to create predictive tools with superior accuracy and provide insight into its nonlinear materials science aspects. Machine learning is a powerful tool that can unravel underlying patterns in complex data. Accordingly, this study endeavors to employ state-of-the-art machine learning techniques to predict the compressive strength of UHPC using a comprehensive experimental database retrieved from the open literature consisting of 810 test observations and 15 input features. A novel approach based on tabular generative adversarial networks was used to generate 6513 plausible synthetic data for training robust machine learning models, including random forest, extra trees, and gradient boosting regression. While the models were trained using the synthetic data, their ability to generalize their predictions was tested on the 810 experimental data thus far unknown and never presented to the models. The results indicate that the developed models achieved outstanding predictive performance. Parametric studies using the models were able to provide insight into the strength development mechanisms of UHPC and the significance of the various influential parameters.

## 1. Introduction

The practical applications of concrete are dependent upon its rheological, mechanical, and durability properties, which in turn are affected by multiple factors including cementitious materials, chemical admixtures, aggregates type and grading, water-to-binder ratio, fibers and other inclusions, curing conditions (temperature and relative humidity), etc. [1,2,3]. Ultra-high-performance concrete (UHPC) has been developed to achieve very high compressive strength along with superior ductility and durability. Its mechanical properties are extremely sensitive to the particle packing density, mixture components, and curing conditions [2,3,4,5]. To produce UHPC with very high compressive strength, high cement content, low water-to-binder (w/b) ratio, fine powders (quartz, silica fume, etc.), well-graded aggregates, and high-range water-reducing admixtures are deployed to achieve superior particle packing density and lowest porosity, while assuring adequate flow and consolidation.

Several researchers in recent decades have explored the mechanical properties of UHPC made with diverse ingredients and mixture proportions [1,2,3,4,5,6]. In particular, the inclusion of eco-efficient supplementary cementitious materials (SCMs), such as fly ash (FA) and ground granulated blast slag furnace (GGBFS), have attracted extensive attention among researchers and engineers [7,8,9,10]. Despite this abundant research, the effect of the inclusion of SCMs on the compressive strength of UHPC has not yet been analyzed systematically. For instance, Alsalman et al. [11] and Wu et al. [12] observed that the replacement of cement by FA led to increased compressive strength of UHPC, whereas contradictory results were reported by Randl et al. [7]. Moreover, there have been various studies indicating that partial replacement of cement by GGBFS reduced the compressive strength of UHPC. For instance, Wang et al. [4] reported that the compressive strength of mixtures incorporating GGBFS as partial replacement for Portland cement was reduced by up to 20%. Randl et al. [7], Zhang et al. [8], and Yang et al. [13] also evidenced reduction in UHPC compressive strength upon using GGBFS as a partial replacement for cement. 

Plain UHPC displays an undesirable brittle behavior, which can hamper its use in many engineering applications [1,14,15]. Thus, various types of fibers, such as steel and synthetic fibers, have been widely used to improve the ductility and impact resistance of UHPC, among which steel microfibers achieved the most promising performance, increasing flexural and tensile strength, and enhanced toughness and impact resistance. Several researchers found that fibers had an insignificant effect on the compressive strength of UHPC, while the degree of cement hydration and particle packing density of the matrix play a more important role in the strength development of UHPC [11,15,16,17]. Such findings magnify the lack of consistent knowledge for predicting the behavior of UHPC incorporating various mixture ingredients, despite the extensive experimental studies in the literature.

Artificial intelligence has proven to be a powerful tool for solving convoluted engineering problems in various fields. Machine learning (ML) algorithms can predict an output target after being trained on a given dataset. For instance, various engineering properties of composite materials have been modeled using powerful ML models, including artificial neural networks (ANNs), support vector machines (SVMs), tree-based ensembles, deep learning (DL), etc. Ben Chaabene et al. [18] conducted an in-depth review of the application of such ML techniques for predicting the mechanical properties of concrete. Moreover, there have been numerous studies which aimed at predicting the mechanical properties of various types of modern concretes, such as recycled aggregate concrete (RCA) [19,20,21,22], high-performance and ultra-high-performance concrete (HPC and UHPC, respectively) [23,24,25,26,27], phase change materials-integrated concrete [28], self-healing concrete [29], etc. For instance, Han et al. [24] used an improved random forest algorithm to predict the compressive strength of HPC. They deployed a dataset included 1030 compressive strength test observations for HPC made of normal cement and cured under normal conditions. Water, cement, GGBFS, FA, fine aggregates, coarse aggregates, and age were the basic input parameters of the dataset, along with five combined variables appended to predict the compressive strength. These combined variables included ratios of w/b, GGBFS-to-water, FA-to-water, coarse aggregate-to-binder, and coarse aggregate-to-fine aggregate. The developed model had a promising performance in predicting HPC compressive strength. It was recommended to use the absolute mass of mixture components as input features for developing predictive models.

The compressive strength of UHPC was modeled using ANN in a recent study by Abuodeh et al. [30]. They used sequential feature selection and neural interpretation diagram techniques to distinguish those mixture components affecting the performance of the ANN model. Accordingly, they compiled a dataset of 110 UHPC mixture designs to predict the 28-day compressive strength. Although they achieved high predictive accuracy, the small size of their dataset, alongside the limited number of mixture components, warrant further effort to collect a more comprehensive dataset to extend the model robustness and generalization capability. The importance of extensive datasets in developing powerful ML models capable of adapting to new, previously unseen data is widely highlighted in the literature. For instance, Marani and Nehdi [28] developed ML models to predict the compressive strength of concrete incorporating phase change materials using 154 data examples. Despite achieving high accuracy, they posited that expanding the dataset should improve the model generalization capability and provide better insights into the materials science aspects of the problem. Therefore, the collection of pertinent and comprehensive experimental data is of great importance in developing ML predictive tools to better understand the non-linear relationship between different mixture components of UHPC and its compressive strength. Moreover, the inclusion of the curing regime including temperature, relative humidity (RH), and time can provide valuable insight into the strength development of UHPC over time and under various curing conditions.

Considering various UHPC mixture components and the diverse existing experimental data available in the open literature, developing robust predictive tools for modeling the mechanical properties of UHPC and understanding the complex relationships between its mixture components are desirable. The present study creates novel ML models to predict the compressive strength of UHPC based on an extensive dataset of wide-ranging experimental data retrieved from reliable resources in the open literature. Furthermore, a state-of-the-art data generating technique was deployed, for the very first time, to generate UHPC compressive strength synthetic data points for training the ML models. Synthetic data generation can mitigate the problems associated with the limited availability of pertinent experimental data for in-depth and comprehensive analysis of UHPC mixture design. Accordingly, tabular generative adversarial networks (TGAN) were able to generate plausible data for training robust tree-based ensembles including random forest (RF), extra trees (ET), and gradient boosting (GB) for the estimation of UHPC compressive strength. Subsequent sections elaborate on the data collection, fundamentals of the applied ML models, performance evaluation metrics, and discussion of the results. Fundamentals of the applied methods along with the model development steps are further explained in Section 3. A comprehensive parametric study was also carried out to gain profound insights into the influence of UHPC mixture ingredients on its compressive strength. 

## 2. Data Collection 

Creating a comprehensive and reliable dataset is a vital step in developing ML predictive models. For this purpose, an extensive literature review was performed to retrieve data from published research papers. Diverse supplementary cementitious materials (SCMs), fine and ultra-fine aggregates, types of fibers, etc., have been incorporated in UHPC to improve its mechanical and durability properties. Therefore, there are many input features that could be considered for an ML model to forecast the compressive strength of UHPC. Considering the numerous experimental studies that used such materials in UHPC mixture designs, along with several curing regimes, a large dataset comprising various mixture components was initially collected. However, to consolidate the proposed predictive framework, the dataset was narrowed down to UHPC mixtures incorporating the most frequently used ingredients. Additionally, only the temperature (T) and relative humidity (RH) were considered as curing conditions. Thus, a dataset consisting of 912 test observations was constructed to estimate the compressive strength of UHPC. This dataset was further preprocessed to eliminate outliers and data examples with missing input values. After preprocessing, 810 test observations and 15 input features were assigned as the final dataset. All the data were collected from research published in respected forums [4,5,6,7,8,11,12,13,16,31,32,33,34,35,36,37,38,39,40,41,42,43,44,45,46,47,48,49]. 

The following assumptions were made in collecting the data: (i) The dosage (absolute mass) of the mixture components for a unit volume of UHPC was collected; (ii) the physical properties of mixture components such as the density and particle size distribution were not included in the final dataset; (iii) only steel fibers were considered in the data collection, and other types of fibers were discarded; the physical and mechanical properties of steel fibers were not included; and (iv) the curing temperature (T) and relative humidity (RH) were considered as the curing conditions. 

The selection of input features was performed considering findings in pertinent experimental studies or previous ML modeling of cementitious composites. For instance, the physical properties of steel fibers such as diameter and length were not included as input features due to their confirmed insignificant effect on compressive strength [14,15]. For instance, Abuodeh et al. [30] used the absolute mass of steel fibers alone in their predictive model. Table 1 presents the variables of the dataset along with their designations. The developed dataset is among the largest available on UHPC mixture designs. Abuodeh et al. [30] used 110 data samples for their ML modeling of UHPC. Qu et al. [50] used 162 data examples on the compressive strength of UHPC. Abellán-García [51] collected 717 data points from the literature along with 210 experimental data from laboratory testing to construct a dataset with 927 observations. After outlier detection, their final number of data points used for training and testing was reduced to 827. The final dataset used for the model development in the present study is presented in Tables S1 and S2 of the supplementary materials. Table S1 presents the input variables of the dataset as well as their designation and units, while Table S2 reports the final dataset used in this study.

## 3. Model Development

This study deployed state-of-the-art machine learning (ML) algorithms to predict the compressive strength of UHPC. For this purpose, a tabular generative adversarial network (TGAN) approach was implemented, for the very first time, to generate a significant amount of synthesized data for training robust and generalized ML models including random forest (RF), extra trees (ET), and gradient boosting (GB), as discussed below.

### 3.1. Machine Learning Fundamentals 

Numerous research studies aimed at estimating the mechanical properties of different types of concrete using ML techniques. Several models demonstrated superior predictive performance owing to their ability to learn the data and its underlying patterns and propose data-driven recommendations and estimates. Artificial neural networks (ANN), fuzzy logic, Gaussian processes, and tree-based ensembles are among the most widely used algorithms [18,28,29,52]. In conventional procedures, the dataset is randomly divided in subsets with one dataset used for training and the other for testing the ML model. Accordingly, regardless of the size of the dataset, about 70–80% of the available data is allocated to the training task, and thus only 20–30% of the data could be employed to verify and test the performance of the model [18,52]. Considering the typically small sizes of datasets available for material processing problems, the small portion of data allocated for testing raises concern about the accuracy, robustness, and generalization capacity of the developed models for future new data previously unseen to the model [18,28,53,54]. To mitigate such problems in ML modeling of compressive strength of UHPC, the present study aims at investigating the application of the new TGAN algorithm to generate a large amount of synthetic data for training ML models. Subsequently, ML models were trained to predict the compressive strength of UHPC by means of both original and synthesized data. The deployed models are briefly outlined below.

#### 3.1.1. Tabular Generative Adversarial Networks (TGAN)

ML models are highly dependent on the adequacy and reliability of data, especially in advanced and complicated applications performed by intricate techniques such as deep learning. However, collecting enough data could be a major challenge for several reasons, such as the high associated costs. For instance, generating new UHPC mixture design data is laborious, costly, and time-consuming, especially considering the multitude of mixture design parameters involved. In addition, making a very large amount of UHPC wastes material and is not eco-efficient. 

The generative adversarial networks (GANs) was first proposed by Goodfellow et al. [55] for generating plausible “fake data” from a target distribution. Thereafter, several studies have been conducted to stabilize the training of GAN and improve its data generating performance. GAN consists of two networks named generator (G) and discriminator (D). The generator synthesizes “fake data”, while the discriminator predicts the probability that the generator’s output is real rather than fake. Both compete in a minmax game of making the generator fool the discriminator in distinguishing whether the data is sampled from the real distribution [55,56,57]. GANs have been widely utilized in computer vision problems, such as generating high-quality and realistic-looking images. Several versions of GAN have been developed for specific tasks, such as conditional GAN (CGAN), Wasserstein GAN (WGAN), cycle GAN (CycleGAN), tabular GAN (TGAN), etc. [57,58,59,60]. Xu and Veeramachaneni [57] developed TGAN to generate plausible synthesized tabular data having multinomial/discrete and continuous variables. They employed long short-term memory (LSTM) neural networks as the generator and multi-layer perceptron (MLP) as the discriminator [57]. The generator is trained using an Adam optimizer to optimize the loss function where the Kullback–Leibler (KL) divergence is added to the loss function as follows [57]:

(1) $${ℒ}_{G}=-E_{Z∼N\left(0,1\right)}\log D\left(G\left(𝓏\right)\right)+\sum_{𝒾=1}^{{𝓃}_{𝒸}}KL({𝓊}_{𝒾}^{\prime },{𝓊}_{𝒾})+\sum_{𝒾=1}^{{𝓃}_{D}}KL({𝒹}_{𝒾}^{\prime },{𝒹}_{𝒾}),$$

where ${ℒ}_{G}$ is the loss function of the generator, *D* is the discriminator, *G* is the generator, ${𝓃}_{𝒸}$ is the number of continuous variables, ${𝓃}_{D}$, is the number of discrete variables, ${𝓊}_{𝒾}$ and ${𝒹}_{𝒾}$ are real data, and ${𝓊}_{𝒾}^{\prime }$ and ${𝒹}_{𝒾}^{\prime }$ are fake data. More details on the TGAN structure can be found in [57]. TGAN can mimic the distribution of single table data having numerical and categorical variables, and thus is a powerful method to generate synthetic data for material science applications compared to other data generation methods, such as autoencoders. The present study deploys the TGAN library in Python developed by Xu and Veeramachaneni [57], which has demonstrated superior performance in tabular data generation using several well-known datasets in the open literature. Due to the use of deep LSTM and MLP networks as the generator and discriminator, TGAN has several parameters and hyperparameters that impact the quality of the synthesized data. Table 2 presents the optimum parameters obtained after an extensive trial and error approach to achieve high quality synthetic data for training ML models. An Adam optimizer was selected as the optimizer in the current study. 

#### 3.1.2. Tree-Based Ensembles

The classification and regression trees (CART) algorithm is among the most widely implemented ML models for classification and regression problems. The fundamental idea of the CART decision tree is to split a complicated prediction task into less complex processes. CART was proposed by Brieman et al. [61] as a non-parametric model for constructing meaningful relationships within the input data to accurately predict the output. Thereafter, ensemble models were introduced to enhance the prediction accuracy of the model, while mitigating the associated risks of over-fitting [28,62,63]. Bagging and boosting have proved to be successful methodologies for developing well-known tree-based ensembles, including random forest (RF), extra trees (ET), gradient boosting (GB), etc. 

**Figure 1:**
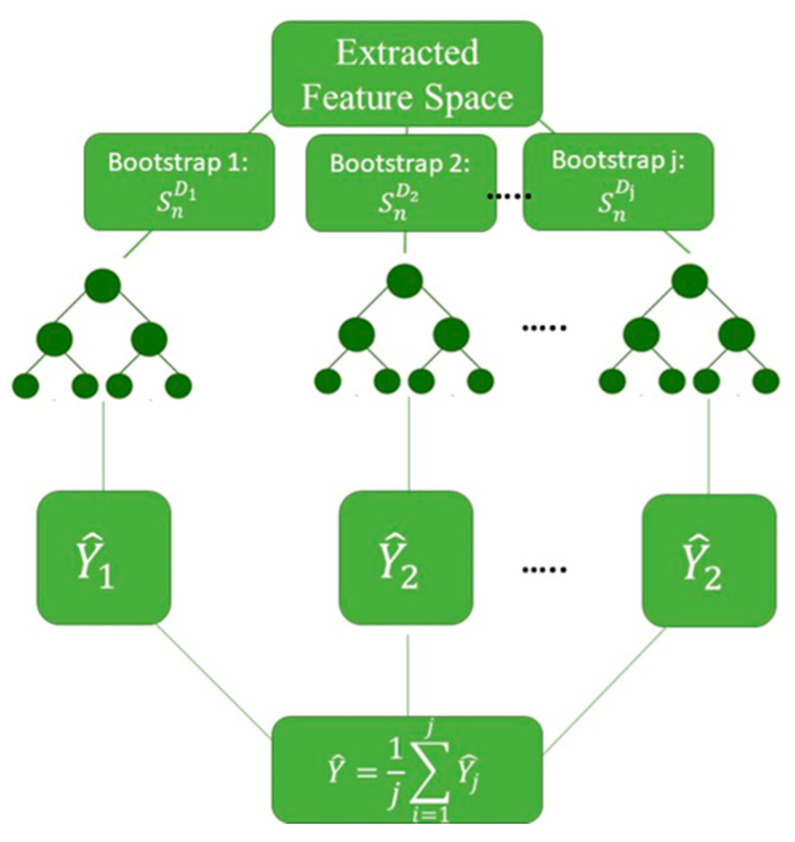
Schematic structure of a random forest regression model.

RF is one of the extensions of the CART algorithm and has yielded promising results in several regression problems. The RF model generates numerous decision trees such that the growth of each tree is controlled by a randomized subset of predictors. RF is an ensemble technique that combines such decision trees by means of a “Bagging” algorithm. Accordingly, a subset of features is sampled in a random fashion for each individual decision tree. This sample is referred to as “bootstrap”. If *X* represents the input vector containing m features as $X=\left\{x_{1},\;x_{2},\;x_{3},\;\ldots ,\;x_{m}\right\}$, *Y* represents the target, and $S_{n}$ represents the dataset, including *n* data examples, as $S_{n}=\left\{\left(X_{1},\;Y_{1}\right),\;\left(X_{2},\;Y_{2}\right),\;\left(X_{3},\;Y_{3}\right),\;\ldots ,\;\left(X_{n},\;Y_{n}\right)\right\}$; the bagging algorithm implements the decision tree algorithm to multiple bootstrap samples, $\left(S_{n}^{D_{1}},\;S_{n}^{D_{2}},\;S_{n}^{D_{3}},\;\ldots ,\;S_{n}^{D_{j}}\right)$. Consequently, *j* prediction trees are constructed to estimate the output, *Y*. These predictions can be expressed as: ${\hat{Y}}_{1}=\hat{h}\left(X,\;S_{n}^{D_{1}}\right)$, ${\hat{Y}}_{2}=\hat{h}\left(X,\;S_{n}^{D_{2}}\right)$, ${\hat{Y}}_{3}=\hat{h}\left(X,\;S_{n}^{D_{3}}\right)$, …, ${\hat{Y}}_{j}=\hat{h}\left(X,\;S_{n}^{D_{j}}\right)$, where $\hat{Y}$ is the estimation by each decision tree, $\hat{h}$. Figure 1 depicts the schematic framework of a decision tree algorithm. The predicted outputs of all trees are averaged to aggregate the predictions as follows: $\hat{Y}=\frac{1}{j}\overset{j}{\underset{i=1}{\sum }}\hat{Y_{j}}$. 

The extra trees (ET) algorithm is an extension of RF with some modifications to reduce the variance of the trained model. Like RF, ET deploys a subset of features selected randomly to train the predictors, as mentioned earlier. Nevertheless, ET randomly acquires the best features for splitting the nodes of the decision tree, in contrast to RF, which selects the most discriminative splits. For this reason, ET is also referred as extremely randomized trees. Another major difference between these two algorithms is that contrary to RF, which utilizes bootstraps to train the estimators, ET uses all the training set for training the predictors. Although this approach could lead to reduction of the variance of the model, it may result in a slight increase in bias [62,64,65,66]. 

In contrast to the RF and ET models that employ a bagging technique, the GB method is based on a boosting approach to amalgamate multiple weak learners for constructing a robust predictor [28,67,68]. Equation (2) presents the stage-wise approach adopted in GB for training additive models. In this model, ${𝒽}_{m}\left(x\right)$ are the weak learners, which are regression decision trees. The GB model combines *m* weak learners such that a new estimator is added to the model upon each iteration. Furthermore, a controlling parameter called “learning rate” is applied to the training of the GB model to limit the contribution of each single decision tree in forecasting the output, as shown in Equation (3). This can help reduce over-fitting of the model [28,67,68,69].

(2) $${ℱ}_{m}\left(x\right)={ℱ}_{m-1}\left(x\right)+{𝒽}_{m}\left(x\right)$$

(3) $${ℱ}_{m}\left(x\right)={ℱ}_{m-1}\left(x\right)+\alpha {𝒽}_{m}\left(x\right)$$

For interested readers, mathematical details of the RF, ET, and GB models are provided elsewhere [63,64,65,66,67,68]. In this study, the scikit-learn package in Python was utilized to construct the models [64].

### 3.2. Performance Evaluation

Assessing the performance of ML models in the training and testing phases is a crucial step to ensure that the model delivers satisfactory performance for future unseen data in terms of accuracy, robustness, and generalization capability. Purposefully, statistical indicators could be employed to evaluate the error of ML models in predicting the target. In this study, the mean absolute error (*MAE*), root mean squared error (*RMSE*), and coefficient of determination (*R*^2^) were used to evaluate the prediction accuracy of each individual model as follows:

(4) $$RMSE=\sqrt{\frac{1}{m}\overset{m}{\underset{i=1}{\sum }}{\left(Y_{i}-{\hat{Y}}_{i}\right)}^{2}}$$

(5) $$MAE=\frac{1}{m}\overset{m}{\underset{i=1}{\sum }}\left|Y_{i}-{\hat{Y}}_{i}\right|$$

(6) $$R^{2}=1-\frac{\sum_{i=1}^{m}{\left(Y_{i}-{\hat{Y}}_{i}\right)}^{2}}{\sum_{i=1}^{m}{\left(Y_{i}-\bar{Y}\right)}^{2}},$$

To evaluate the performance of the data generated by traditional GAN, a visual inspection of generated data was carried out to distinguish the fake data. However, this is a qualitative and biased procedure not applicable to tabular data [56,70,71]. Therefore, using quantitative metrics is inevitable to assess whether the synthetic data is credible. Hence, researchers proposed to evaluate the performance of ML models trained with real/synthetic data as a quantitative performance assessment. For instance, El Kababji and Srikantha [70] used a neural network to test whether the synthetic data resembled the real data. Since one of the major objectives of the current study was to generate plausible synthetic data for training ML models, a quantitative approach was adopted to evaluate the validity of synthesized data as per the recommendations of Fekri et al. [56] and Esteban et al. [71]. In this approach, the credibility of synthetic data is inferred by the performance of ML models. In conventional procedures, ML models are trained on 70–80% of the available data, and the remaining 20–30% are allocated for testing the model performance. For real data, this approach is referred to as train on real, test on real (TRTR). Although this approach does not provide a means of assessing synthetic data, it allows one to compare the accuracy of models trained with real and synthetic data. This approach could also be adopted for synthetic data, referred to as train on synthetic, test on synthetic (TSTS). This could demonstrate whether the generated “fake data” resembles the real data, and thus the performance of models trained with them are comparable [56].

In addition to the TRTR and TSTS approaches, the reliability of the synthetic data could be evaluated by adopting another modeling approach. The ML models are once trained with the entire synthesized dataset and tested on the entire real dataset. This approach is referred to as train on synthetic, test on real (TSTR). The reverse of TSTR is train on real, test on synthetic (TRTS). Both TSTR and TRTS models are used to evaluate whether TGAN could generate plausible synthetic data, and thus training ML models on synthetic data could achieve promising performance [56,71]. Ultimately, random forest (RFR), extra trees (ETR), and gradient boosting (GBR) regression models were developed separately considering the TRTR, TSTR, TRTS, TSTS approaches. The predictive performance of each individual model was assessed using the statistical indicators explained earlier. After evaluating the credibility of synthesized data, the TSTR models were selected as the final models for this study to predict the compressive strength of UHPC mixtures using all data examples.

## 4. Results and Discussion

This section scrutinizes the results obtained from the machine learning (ML) modeling of the UHPC compressive strength based on the extensive dataset collected from the open literature and TGAN synthetic data used for model training. ML model performance is then compared to previous pertinent work in the literature. 

### 4.1. Machine Learning Modeling

First, a tabular generative adversarial network (TGAN) model was developed to generate plausible synthetic data. To achieve most realistic synthetic data, it is crucial to tune the parameters of the TGAN model. Table 2 presents the parameters and hyperparameters of TGAN implemented in the current study. For better convergence of the TGAN model, four input features including the fiber content, relative humidity (RH), temperature (T), and age were considered as discrete variables; 7000 synthetic data were generated, of which 6513 were used for model development after preliminary preprocessing. Table 3 compares the statistical features of the real data to that of the synthetic data generated by the TGAN model for the continuous input features. TGAN was able to mimic the distribution of the real data and sampled plausible data examples such that the statistical characteristics of both datasets were in good agreement.

To evaluate the credibility of synthetic data, the RFR, ETR, and GBR models were developed considering the approaches described earlier. Initially, all three applied ML models were first tuned using 70% of the real dataset (i.e., training data) using a five-fold cross-validation approach as a conventional procedure in ML modeling [18,28,72]. Table 4 presents the tuned parameters of the RFR, ETR, and GBR models. Next, the same parameters were used to create the models based on TRTR, TSTR, TRTS, and TSTS approaches. TRTR models are used as a benchmark for evaluating the quality of the synthetic data. In other words, the predictive accuracy of the ML models trained with synthetic data should be similar to that of the models trained with real data to validate the reliability of synthetic data. Accordingly, the predictive accuracy of each single model was evaluated using the statistical indicators MAE, RMSE, and $R^{2}$. 

**Figure 2:**
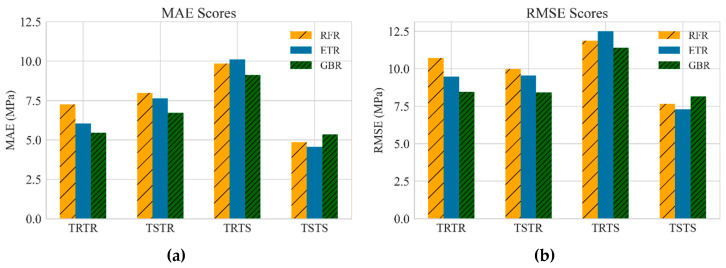
Comparison of performance of developed models (**a**) MAE score, and (**b**) RMSE score.

**Figure 3:**
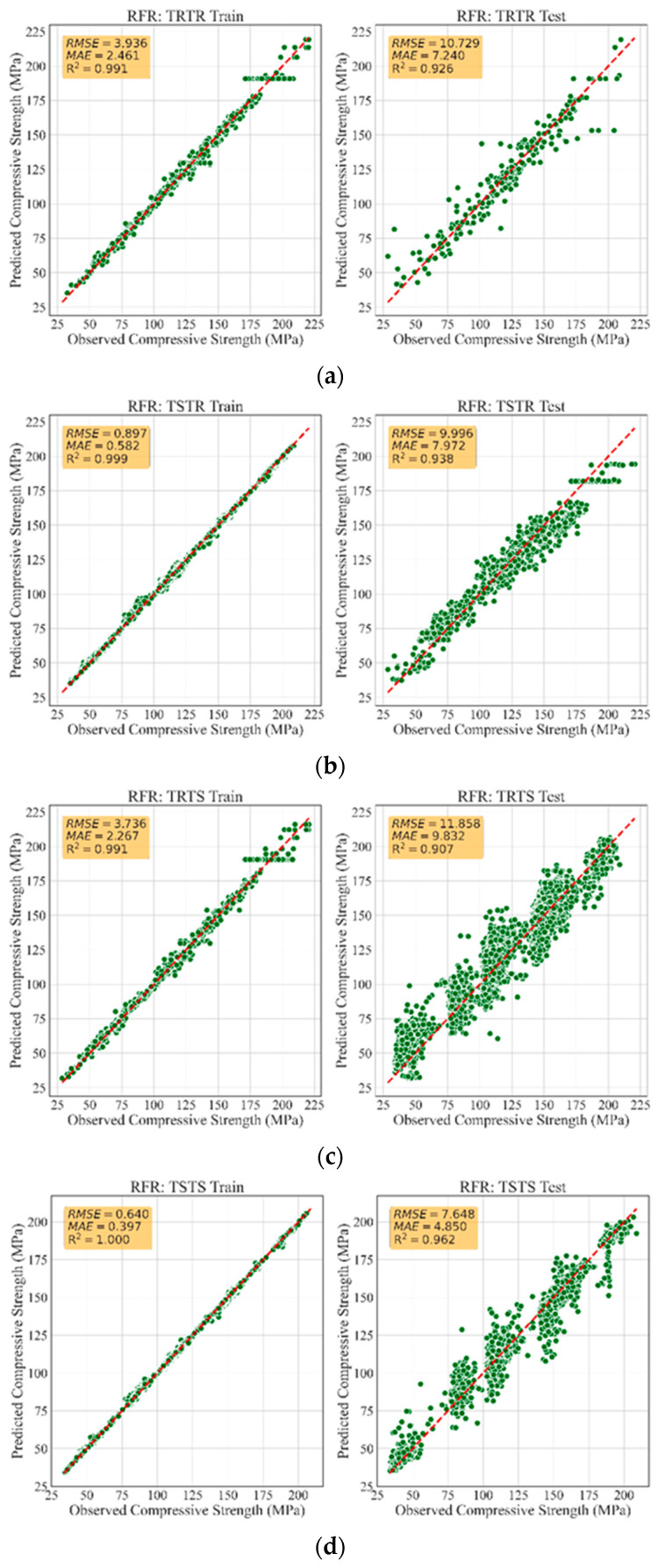
Training and testing performance of RFR model: (**a**) TRTR; (**b**) TSTR; (**c**) TRTS, and (**d**) TSTS.

**Figure 4:**
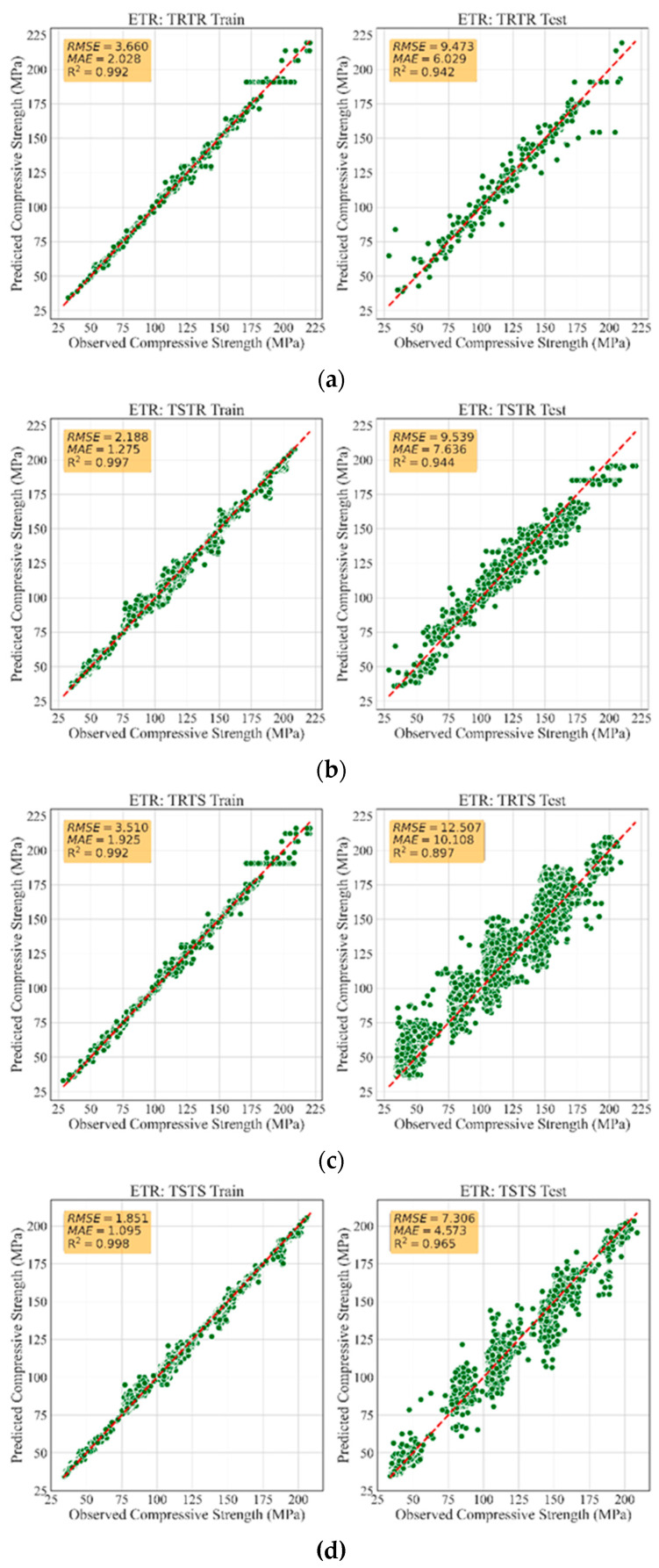
Training and testing performance of ETR model: (**a**) TRTR; (**b**) TSTR; (**c**) TRTS, and (**d**) TSTS.

**Figure 5:**
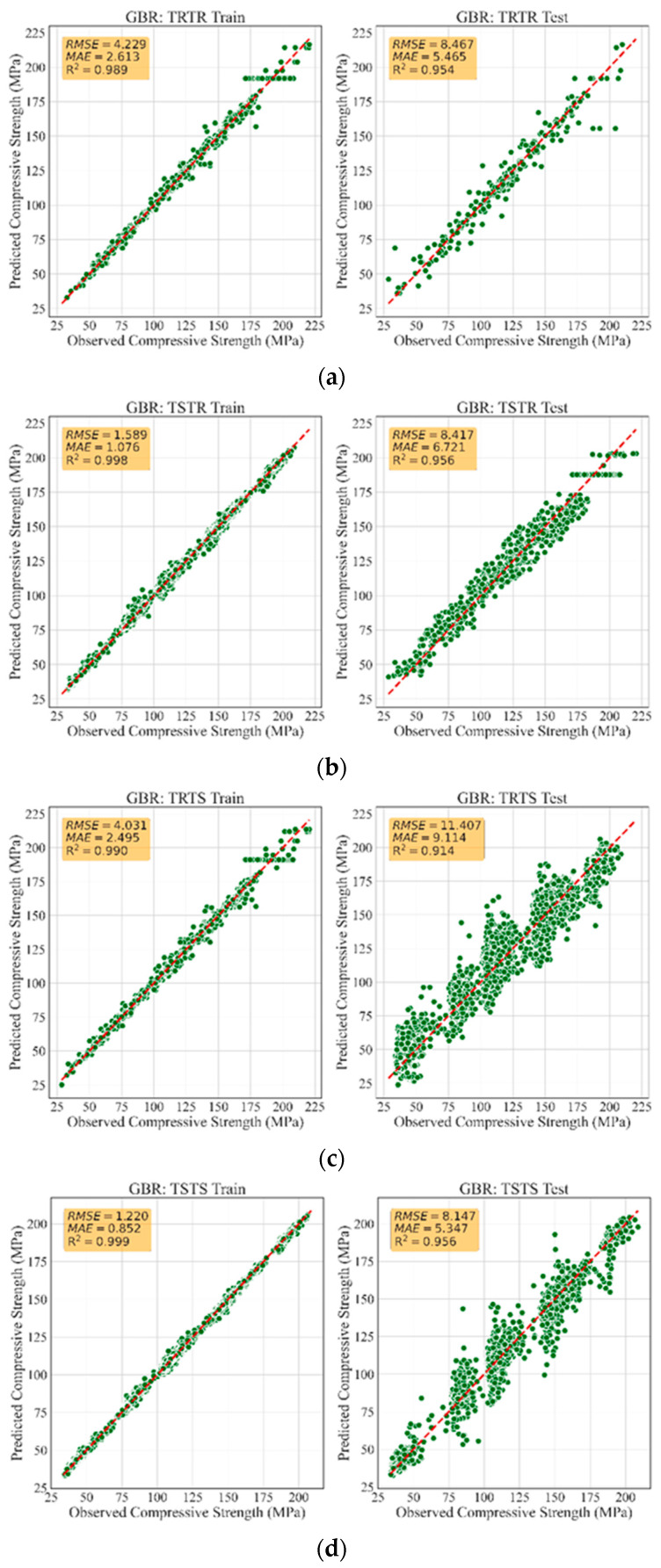
Training and testing performance of GBR model: (**a**) TRTR; (**b**) TSTR; (**c**) TRTS, and (**d**) TSTS.

Table 5 summarizes the performance evaluation of all developed models. Accordingly, the models trained with synthetic data had similar performance to those trained with real data, demonstrating that the TGAN model was able to generate high-quality synthetic data. In fact, the models trained with synthetic data were robust and generalized such that they accurately recognized the patterns in the real data and accurately predicted the compressive strength of UHPC with small error. For instance, training RFR, ETR, and GBR models with synthetic data led to $R^{2}$ values as high as 0.93, 0.94, and 0.95, respectively, when tested with all the real data, i.e., the TSTR approach. Such accuracies were very similar to the accuracies of models trained with real data. This highlights that the TGAN model was able to adequately learn the distribution of the real data and sample realistic data. Figure 2 depicts a comparison between the MAE and RMSE values for all models. It can be observed that training the models with synthetic data was performed successfully, such that low MAE and RMSE values were achieved. Such error values were lower than the errors reported in similar studies [30,51]. This demonstrates the significant potential of TGAN to generate credible data for training powerful and generalized ML models. Figure 3, Figure 4 and Figure 5 illustrate the prediction accuracy of RFR, ETR, and GBR models using the TRTR, TSTR, TRTS, and TSTS approaches.

**Figure 6:**
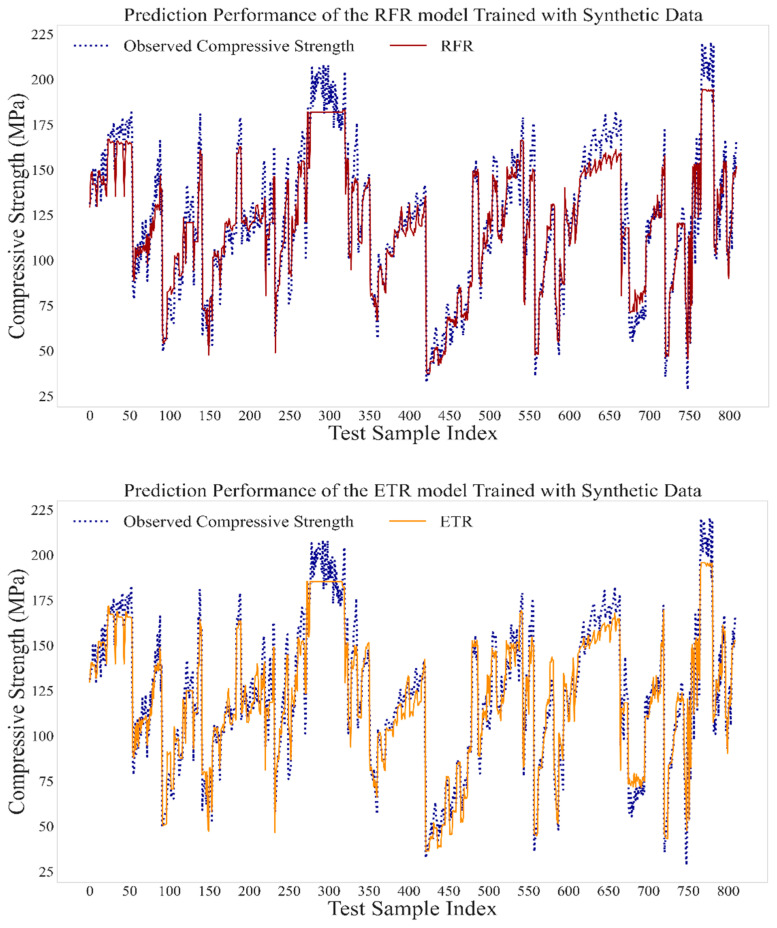

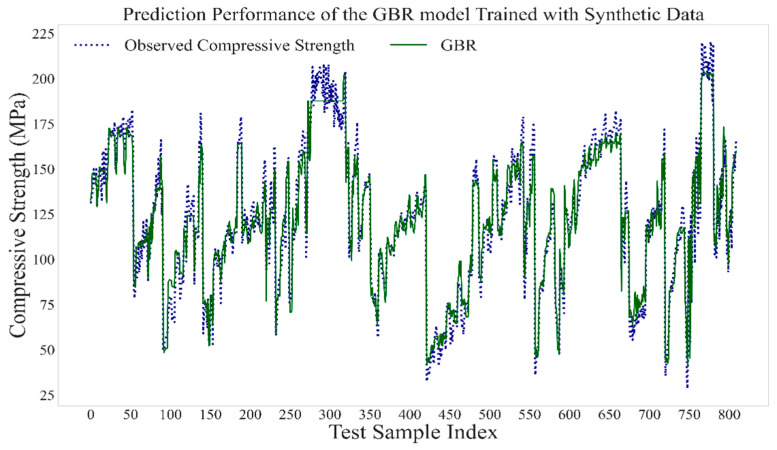
Prediction performance of RFR, ETR, and GBR models using the TSTR approach.

After demonstrating the reliability of synthetic data for training ML models by achieving high accuracy in all approaches, the TSTR approach was selected as the main modeling approach in this study. Figure 6 indicates the compressive strength for data examples in the real dataset along with the predicted compressive strengths by RFR, ETR, and GBR models using the TSTR approach. It can be observed that the models precisely predicted the compressive strength of UHPC. Ultimately, all three ML algorithms adopted in this study achieved satisfactory prediction performance and can be generalized for future unseen data and extensive parametric analysis of mixture components. For this purpose, a voting regressor composed of RFR, ETR, and GBR models was employed to perform parametric analysis, as described below.

### 4.2. Comparing with Other Studies

One of the main goals of the current study was to generate and use synthetic data to train powerful ML models to predict the compressive strength of UHPC using the TSTR approach. The validity of the synthetic data, and thus TSTR models, was first proved by comparing its performance versus that of the TRTR and TRTS models, which were trained with real data, as explained earlier. This approach allowed for training of the models and synthetic data but testing them on all the real data points of a large and wide-varying experimental dataset collected from the literature. This can further validate the generalization capability of the TSTR models, as they are tested with a large number of real experimental data. In this regard, the 810 data observations in the real dataset were used to test the RFR, ETR, and GBR models using the TSTR approach as the final models considered in this study.

Abuodeh et al. [30] collected 110 data points and allocated 70% of this data for training, 15% for validation, and 15% for testing, which means that only 17 data points were used for testing their developed ANN model. In another study, Qu et al. [50] used 166 experimental data for training and testing an ANN model to predict the effect of steel fibers on the compressive strength of UHPC; 80% of the dataset (133 data points) was used for training, while only 33 data points were used for testing the model. Abellán-García [51] collected a large dataset consisting of 837 datapoints to predict the compressive strength of UHPC; 209 data points (25% of the dataset) were used to test the developed multi-layer perceptron (MLP) model. Therefore, in the present study, a significantly much larger test dataset was utilized to evaluate the prediction accuracy and generalization capability of the developed models than in previous studies. Additionally, the effect of the curing condition and the age of specimens at testing were included in the dataset, in contrast to previous studies that only studied the 28-day compressive strength of UHPC cured under a standard condition. Yet, the results of the current study are superior compared to those of the aforementioned studies. The $R^{2}$ achieved herein for the testing dataset was 0.93, 0.94, and 0.95 for RFR, ETR, and GBR models, respectively, when using the TSTR approach. Conversely, Abuedeh et al. [30] and Abellán-García [51] achieved $R^{2}$ values of 0.80 and 0.81, respectively. The MAE and RMSE values of the Abellán-García model were 8.958 MPa and 9.925 MPa, respectively, while the current study achieved lower MAE and RMSE values at 6.72 MPa and 8.41 MPa, respectively. Qu et al. [50] did not report MAE and RMSE values but reported $R^{2}$ of 0.99 for the testing dataset. Such findings emphasize that using the TGAN model for generating synthetic data can boost the performance of ML models.

## 5. Parametric Analysis

The robust predictive performance of the developed ML models along with the numerous credible data points generated by the TGAN model encourage a comprehensive parametric analysis to be conducted to better understand the effects of mixture components on the compressive strength of UHPC. Investigating the effects of various dosages of different mixture ingredients in laboratory experiments is laborious, costly, time-consuming, and associated with a negative environmental footprint. Thus, using robust and well-trained ML models can resolve such problems and broaden the outlook of UHPC materials science.

Accordingly, several case studies for parametric analysis were designed with respect to the UHPC research trends in most recent years. The replacement of cement with eco-efficient SCMs such as slag (S) and fly ash (FA) has attracted vast attention. Using such SCMs can mitigate the carbon footprint of UHPC production, whilst offering satisfactory mechanical properties. Hence the effect of the replacement of cement with S or FA at mass percentages varying from 0 to 50% was assessed. For this purpose, two control mixture designs along with two case studies were considered, as outlined in Table 6. Each case study was applied on both control mixtures. For each control mixture design, the cement content was taken as 750 kg/m^3^, and only silica fume was used as the SCM. Moreover, the analysis explored the effect of three different SF contents along with five water-to-cement ratios (W/C) on the compressive strength of UHPC. The main constraint considered in the design of the parametric analysis was having a unit volume for all mixture designs.

Since all developed ML models demonstrated satisfactory performance, a voting regressor was adopted to predict the compressive strength of UHPC by aggregating predictions of the RFR, ETR, and GBR models. A voting model is an ensemble meta-estimator that combines several base regression models and trains each on the entire training dataset, which was the TGAN generated synthetic data in the present study. Afterwards, it averages each single estimation to yield a final predicted target [64]. Ultimately, the 28-day compressive strength of the mixtures hypothetically cured under a standard condition (T = 23° C and RH = 100%) was predicted using the voting regressor.

### 5.1. Replacing Cement with Slag

**Figure 7:**
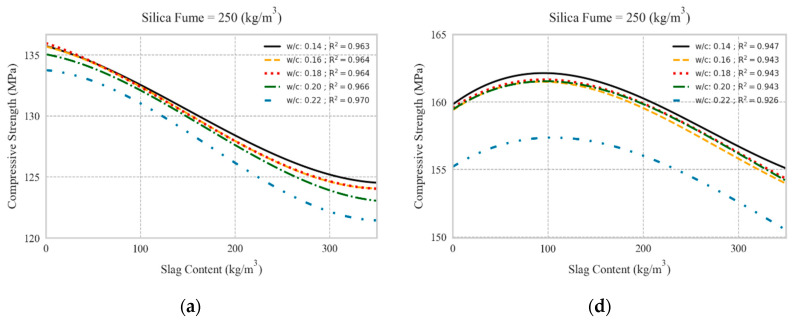

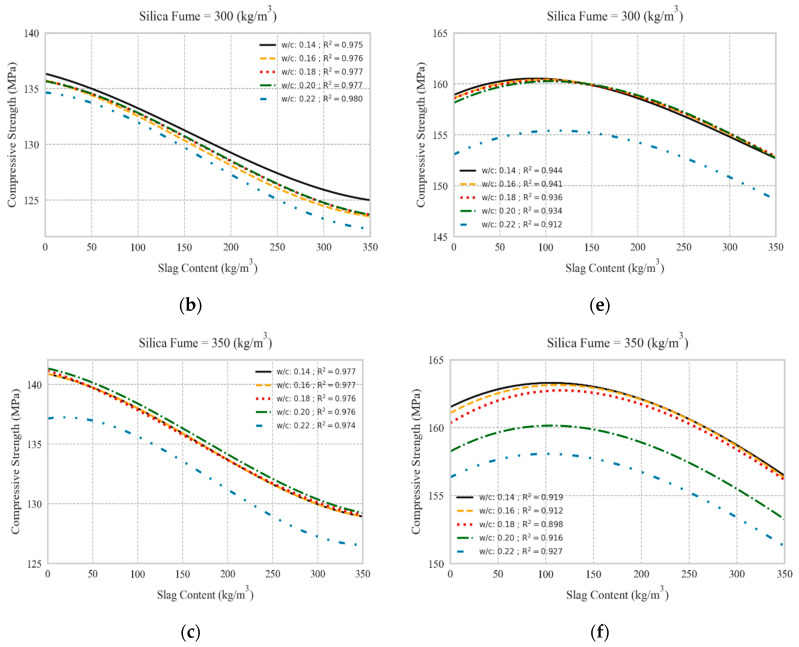
Effect of slag partial replacement for cement on compressive strength: (**a**–**c**): without steel fiber having 250, 300, and 350 kg/m^3^ silica fume, respectively; (**d**–**f**): with 2% vol. steel fibers having 250, 300, and 350 kg/m^3^ silica fume, respectively.

Figure 7 illustrates the influence of different levels of slag partial replacement for cement on the compressive strength of UHPC. In UHPC mixtures with no steel fibers, increasing the slag content slightly decreased the 28-day compressive strength, such that when the slag inclusion was 350 kg/m^3^, the compressive strength reduction was less than 10%. A similar trend was observed for different SF contents, as well as different W/C ratios considered in this study. Lower W/C ratios and higher SF contents resulted in higher compressive strengths, as expected. On the other hand, when the UHPC mixtures incorporated 2% by volume of steel fibers (equivalent to 156 kg/m^3^), the compressive strength was generally higher compared to that of mixtures with no steel fibers. Moreover, the replacement of cement with slag at lower dosages (up to 150 kg/m^3^) slightly improved the compressive strength, while at higher dosages (350 kg/m^3^) the compressive strength was decreased by less than 10%. In other words, the reduction of compressive strength in mixtures with steel fiber was less than that for mixtures without steel fibers. A similar trend was evidenced regarding various SF contents and W/C ratios, as shown in Figure 7. Overall, the results suggested that the partial replacement of cement with slag maintained desired compressive strength of UHPC mixtures.

### 5.2. Replacing Cement with Fly Ash

**Figure 8:**
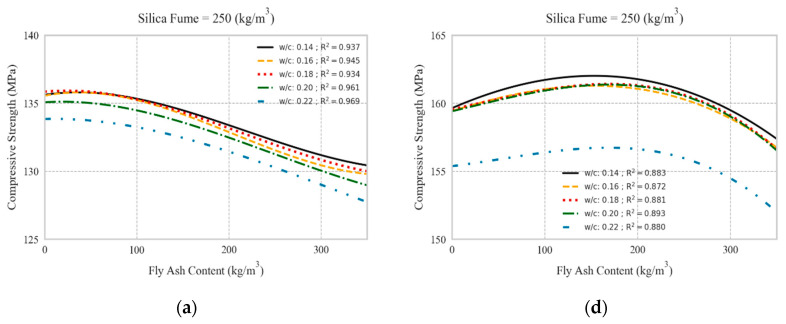

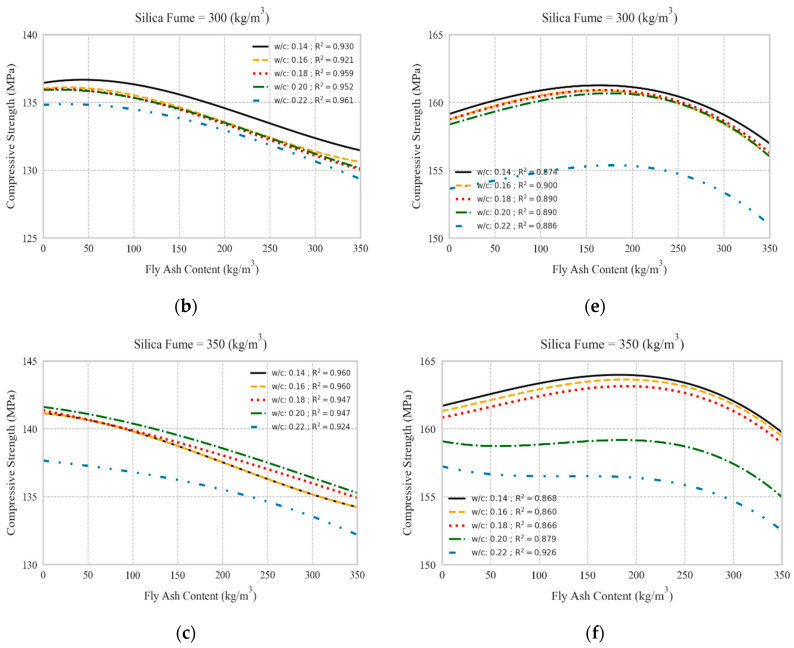
Effect of fly ash partial replacement for cement on compressive strength: (**a**–**c**): without steel fiber having 250, 300, and 350 kg/m^3^ silica fume, respectively; (**d**–**f**): with 2% vol. steel fibers having 250, 300, and 350 kg/m^3^ silica fume, respectively.

**Figure 9:**
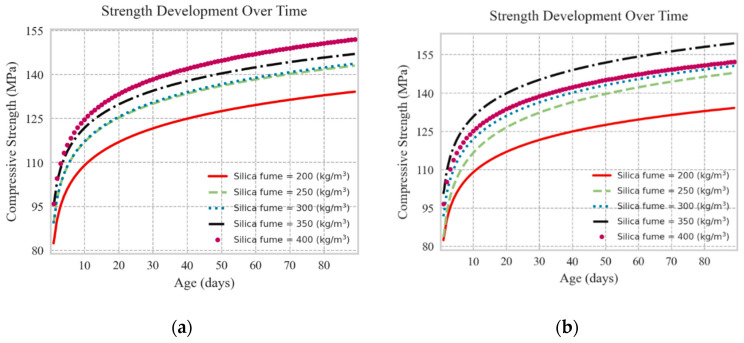
Strength development of UHPC mixtures over time; (**a**) cement content: 750 kg/m^3^, and (**b**): cement content 1000 kg/m^3^.

The effect of FA inclusion as partial replacement for cement on the compressive strength of UHPC mixtures with and without steel fibers is illustrated in Figure 8. The replacement of cement with FA led to insignificant reduction in compressive strength, like the trend observed for slag. However, when using FA with higher SF contents and W/C ratios, the reduction of compressive strength was slightly larger compared to that at lower SF content and W/C ratios. Moreover, in UHPC mixtures incorporating steel fibers, FA partial cement replacement at dosages of up to 200–250 kg/m^3^ marginally enhanced the compressive strength, whereas FA levels beyond this threshold decreased the compressive strength. Like mixtures without steel fibers, the reduction of compressive strength due to replacement of cement with FA was more evident at higher SF content and W/C ratios. Yet, a high compressive strength of UHPC mixtures was still achievable using high FA dosages. Such findings are in agreement with experimental findings reported in the literature [7,8,13,45]. Thus, performing comprehensive parametric analyses using robust and generalized ML models can be a powerful tool for identifying combined effects of parameters on the compressive strength of UHPC. Owing to the inclusion of the age of specimens at the testing time, the effect of time on the strength development of UHPC mixtures could be simulated as well. For instance, the strength development of UHPC mixtures beyond 90 days was depicted for two control mixture designs having cement contents of 750 kg/m^3^ and 1000 kg/m^3^ in Figure 9. It was observed that the models captured the strength development of UHPC mixtures having various silica fume contents over the time. 

## 6. Limitations of the Model

Concrete is a highly heterogenous material, characterized by brittle fracture. Developing predictive models for its mechanical properties based on its fracture process requires thorough understanding of its behavior over a wide range of scales, and quantitative evaluation of multiple parameters governing its micro-and macro-cracking [73]. Several attempts have been made to model the fracture process of concrete using liner-elastic fracture mechanics with the fracture zone surrounded by an elastic region characterized by stress intensity factors (linear) or *J* integrals (nonlinear). This approach was, however, unable to predict the actual fracture behavior of concrete [74]. Generally, it was found that defining unique critical stress intensity factors or *J* integrals and *R* curves was not successful for cementitious materials [74]. Various schemes have thus been developed to model the fracture process zone in concrete using nonlinear fracture models. 

For instance, Kurumatani et al. [75] proposed an isotropic damage model for quasi-brittle materials such as concrete. This damage model was claimed to simulate the strain-softening behavior of concrete without mesh-size dependency. While the application of fracture mechanics to concrete garnered great interest, it has not led to reliable and practical models that can be implemented in design codes and industry applications. The common current practice is rather to rely on empirical models based on regression analysis of existing experimental data.

Moreover, several continuum or discrete models have been proposed to simulate the fracture mechanism of concrete, such as the extended finite element method (XFEM), lattice model, etc. [76,77,78,79,80,81]. For instance, Smith et al. [78] simulated the behavior of UHPC using a lattice discrete particle model using the parameters identified by various quasi-static tests, such as single pull-out, uniaxial compression and strain, triaxial compression, etc. Their findings indicated that the micro-splitting failure due to the hooks at fiber ends with the brittleness of the cement matrix should be taken into account in failure mechanism analysis of UHPC [78]. Such findings suggest the viability of machine learning modeling of the fracture mechanism of concrete using extensive experimental data in future work. Furthermore, fracture mechanics models have been mostly applied to simulate tensile or flexural strength of concrete, along with its ductility and impact behavior [82,83]. Data driven methods can further complement the findings in such studies considering the wide-ranging experimental data in the literature. It is noteworthy that few studies have investigated the fracture mechanism numerically for UHPC incorporating various supplementary cementitious materials and fibers. Thus, more comprehensive research is needed to bridge the knowledge gap found in pertinent experimental data. 

More recently, there has been growing interest in using data driven artificial intelligence models to predict the mechanical properties of concrete. Such methods do not impose a model on the data. The model is rather created through learning algorithms from the structure of the data itself. The more comprehensive the data set, the more successful could be the training of the data driven model, and the more accurate would be the model predictions. Another advantage is that while traditional regression analysis models fail to capture the highly complex and nonlinear relations between the mixture ingredients of materials such as UHPC and its mechanical strength, data driven machine learning algorithms can excel in capturing such a behavior.

Therefore, it should be understood that the model proposed in this study is not a substitute for the meso-scale materials science understanding of concrete, nor does it try to capture the fracture behavior of the material. The model simply learns the relationship between the concrete mixture ingredients and its mechanical strength from existing data examples. When the learning is effective, the model can generalize its predictions to new data examples never presented to the model before. Such a performance is demonstrated in this paper on a large set of experimental data examples. However, if the new data example is outside the scope of the training of the model, it will likely not yield accurate prediction. Moreover, the dataset used in this study does not include data specific to the ductility/brittleness of UHPC mixtures. The compressive strength of UHPC was the only experimental parameter modeled. The analysis of the tensile and flexural strengths along with the ductility of UHPC with respect to its mixture ingredients can be the objective of future work. 

## 7. Conclusions and Future Work

The present study proposes a novel framework for predicting the compressive strength of UHPC using state-of-the-art machine learning models. For this purpose, 810 experimental data points were retrieved from studies in the open literature. A tabular generative adversarial net (TGAN) model was employed to generate credible synthetic data for training the ML models so that the entire real dataset could be used for testing the models. Random forest (RFR), extra trees (ETR), and gradient boosting (GBR) regression models were tuned and trained as the baseline predictors. Based on the results, the following conclusions can be drawn:

- The TGAN can be used to generate plausible synthetic data capable of adequately training powerful and generalized ML models.
- Statistical metrics of *R*^2^ of 0.96 and *MAE* and *RMSE* values of 6.72 MPa and 7.41 MPa, respectively, were achieved for the testing set when the GBR model was trained with synthetic data and tested on the entire real data.
- Such predictive performance is outstanding when compared to that of existing models in the literature, which achieved significantly lower performance.
- A voting regressor assembled of RFR, ETR, and GBR models was used to perform parametric analysis on UHPC mixture designs. These models captured the behavior of UHPC compressive strength upon variation of the mixture components.
- Therefore, these models can be employed to provide practical insights into the mixture design of UHPC for diverse construction applications, providing enhanced predictive capacity at lower cost and in much shorter time.
- The developed models are data driven based on learning from existing data. Thus, they neither offer an alternative to fracture mechanics approaches, nor would be applicable outside the scope of the data set used in training.

## Figures and Tables

**Table 1:** Variables considered in final dataset extracted from literature.

Variable	Designation	Unit	Variable	Designation	Unit
Cement	C	kg/m^3^	Fine aggregate	Sand	kg/m^3^
Silica fume	SF	kg/m^3^	Coarse aggregate	Gravel	kg/m^3^
Slag	S	kg/m^3^	Fiber	Fi	kg/m^3^
Fly ash	FA	kg/m^3^	Superplasticizer	SP	kg/m^3^
Quartz powder	QP	kg/m^3^	Temperature	T	°C
Limestone powder	LP	kg/m^3^	Relative humidity	RH	%
Nano silica	NS	kg/m^3^	Age	Age	days
Water	W	kg/m^3^	Compressive strength	$f_{c}^{\prime }$	MPa

**Table 2:** Parameters and hyperparameters of the tabular generative adversarial network (TGAN) model.

Parameters	Value	Parameters	Value
Number of RNN cell’s in generator	400	Learning rate	0.001
Number of fully connected units in generator	100	Batch size	200
Number of layers in discriminator	2	Number of train epochs	20
Number of units per layer in discriminator	200	Number of steps in epoch	6000

**Table 3:** Statistical comparison of real data and synthetic data.

**-**	**C (kg/m^3^)**		**SL (kg/m^3^)**		**SF** **(kg/m^3^)**		**LP (kg/m^3^)**	
	**Real**	**Synthetic**	**Real**	**Synthetic**	**Real**	**Synthetic**	**Real**	**Synthetic**
**Mean**	737.91	751.11	25.19	21.71	136.99	148.83	41.93	39.15
**STD**	173.46	157.65	74.37	72.75	104.14	105.26	133.13	145.92
**Min**	270.00	342.37	0.00	0.00	0.00	0.00	0.00	0.00
**25%**	620.20	671.04	0.00	0.00	43.70	47.32	0.00	0.00
**50%**	770.50	785.53	0.00	0.00	144.00	190.93	0.00	0.00
**75%**	850.00	853.82	0.00	0.00	219.00	239.64	0.00	0.00
**Max**	1251.20	1266.87	375.00	378.49	433.70	433.70	1058.20	1058.20
**-**	**QP (kg/m^3^)**		FA **(kg/m^3^)**		**NS** **(kg/m^3^)**		**W (kg/m^3^)**	
	**Real**	**Synthetic**	**Real**	**Synthetic**	**Real**	**Synthetic**	**Real**	**Synthetic**
**Mean**	33.27	37.45	26.26	20.29	3.64	2.76	179.89	180.75
**STD**	79.67	82.80	67.46	60.11	7.78	6.62	25.57	23.28
**Min**	0.00	0.00	0.00	0.00	0.00	0.00	90.00	102.36
**25%**	0.00	0.00	0.00	0.00	0.00	0.00	163.00	167.11
**50%**	0.00	0.00	0.00	0.00	0.00	0.00	177.00	176.91
**75%**	0.00	0.00	0.00	0.00	4.00	0.00	192.50	185.61
**Max**	397.00	404.49	356.00	364.81	47.50	46.20	272.60	260.98
**-**	**Sand (kg/m^3^)**		**Gravel** **(kg/m^3^)**		**SP** **(kg/m^3^)**		$f_{c}^{\prime }$ **(MPa)**	
	**Real**	**Synthetic**	**Real**	**Synthetic**	**Real**	**Synthetic**	**Real**	**Synthetic**
**Mean**	995.33	1019.21	154.78	81.66	30.03	31.53	123.13	120.93
**STD**	283.27	272.00	357.57	266.53	13.99	13.09	40.24	38.92
**Min**	0.00	134.88	0.00	0.00	1.10	3.38	28.51	33.64
**25%**	786.40	833.32	0.00	0.00	18.00	21.16	96.00	104.69
**50%**	1021.00	1050.42	0.00	0.00	30.20	32.21	122.30	111.68
**75%**	1231.00	1239.66	0.00	0.00	44.20	44.96	154.28	149.05
**Max**	1502.80	1488.59	1195.00	1154.54	57.00	56.38	220.50	208.71

**Table 4:** Tuned parameters for the employed machine learning (ML) models.

-	Tuned Parameters
**RFR**	n_estimators = 90; min_samples_split = 3; max_depth = 22; max_features = 4
**ETR**	n_estimators = 100; min_samples_split = 3; max_depth = 20; max_features = 10
**GBR**	n_estimators = 85; learning_rate = 0.9; min_samples_split = 2; min_samples_leaf = 5; max_depth = 16, max_features = 9, subsample = 0.49

**Table 5:** Statistical performance indicators for developed models.

Model	TRTR			TSTR			TRTS			TSTS		
	RFR	ETR	GBR	RFR	ETR	GBR	RFR	ETR	GBR	RFR	ETR	GBR
**MAE**	7.24	6.03	**5.46**	7.98	7.63	**6.72**	9.83	10.10	**9.11**	4.85	**4.57**	5.34
**RMSE**	10.73	9.47	**8.47**	9.99	9.54	**8.41**	11.86	12.50	**11.40**	7.46	**7.30**	8.15
$R^{2}$	0.92	0.94	**0.95**	0.93	0.94	**0.95**	0.90	0.90	0.90	**0.96**	**0.96**	**0.96**

**Table 6:** Control mixture designs and case studies for parametric analysis.

Mix Component	Control Mixture 1	Control Mixture 2	Case Study 1	Case Study 2
**Cement**	750	750	Replaced by slag	Replaced by fly ash
**Silica fume**	250	250	Varying: 250, 300, 350	Varying: 250, 300, 350
**Slag**	0	0	Added as replacement of cement	Added as replacement of cement
**Fly ash**	0	0	-	-
**Limestone powder**	0	0	-	-
**Quartz powder**	0	0	-	-
**Nano silica**	0	0	-	-
**Water**	105	105	W/C ratio: 0.14, 0.16, 0.18, 0.2, 0.22	W/C ratio: 0.14, 0.16, 0.18, 0.2, 0.22
**Fine aggregate**	1367.39	1367.39	-	-
**Coarse aggregate**	0	0	-	-
**Fiber**	0	156	-	-

## Supplementary Materials

The following are available online at https://www.mdpi.com/1996-1944/13/21/4757/s1, Table S1: Variables considered in final dataset extracted from literature. Table S2: Final dataset used for machine learning modeling.

## References

[1] Wang D., Shi C., Wu Z., Xiao J., Huang Z., Fang Z. (2015). A review on ultra-high-performance concrete: Part II. Hydration, microstructure and properties. Constr. Build. Mater..

[2] Yoo D.-Y., Banthia N. (2016). Mechanical properties of ultra-high-performance fiber-reinforced concrete: A review. Cem. Concr. Compos..

[3] Zhou M., Lu W., Song J., Lee G.C. (2018). Application of ultra-high-performance concrete in bridge engineering. Constr. Build. Mater..

[4] Wang C., Yang C., Liu F., Wan C., Pu X. (2012). Preparation of ultra-high-performance concrete with common technology and materials. Cem. Concr. Compos..

[5] Yu R., Spiesz P., Brouwers H. (2014). Mix design and properties assessment of ultra-high performance fibre reinforced concrete (UHPFRC). Cem. Concr. Res..

[6] Yu R., Spiesz P., Brouwers H. (2014). Effect of nano-silica on the hydration and microstructure development of ultra-high-performance concrete (UHPC) with a low binder amount. Constr. Build. Mater..

[7] Randl N., Steiner T., Ofner S., Baumgartner E., Mészöly T. (2014). Development of UHPC mixtures from an ecological point of view. Constr. Build. Mater..

[8] Zhang X., Zhao S., Liu Z., Wang F. (2019). Utilization of steel slag in ultra-high-performance concrete with enhanced eco-friendliness. Constr. Build. Mater..

[9] Chen T., Gao X., Ren M. (2018). Effects of autoclave curing and fly ash on mechanical properties of ultra-high-performance concrete. Constr. Build. Mater..

[10] Arora A., Aguayo M., Hansen H., Castro C., Federspiel E., Mobasher B., Neithalath N. (2018). Microstructural packing-and rheology-based binder selection and characterization for Ultra-high-Performance Concrete (UHPC). Cem. Concr. Res..

[11] Alsalman A., Dang C.N., Hale W.M. (2017). Development of ultra-high-performance concrete with locally available materials. Constr. Build. Mater..

[12] Wu Z., Shi C., Khayat K.H., Xie L. (2018). Effect of SCM and nanoparticles on static and dynamic mechanical properties of UHPC. Constr. Build. Mater..

[13] Yang R., Yu R., Shui Z., Gao X., Xiao X., Zhang X., Wang Y., He Y. (2019). Low carbon design of an ultra-high-performance concrete (UHPC) incorporating phosphorous slag. J. Clean. Prod..

[14] Hoang A.L., Fehling E. (2017). Influence of steel fiber content and aspect ratio on the uniaxial tensile and compressive behavior of ultra-high-performance concrete. Constr. Build. Mater..

[15] Larsen I.L., Thorstensen R.T. (2020). The influence of steel fibres on compressive and tensile strength of ultra-high-performance concrete: A review. Constr. Build. Mater..

[16] Liang X., Wu C., Su Y., Chen Z., Li Z. (2018). Development of ultra-high-performance concrete with high fire resistance. Constr. Build. Mater..

[17] Arora A., Yao Y., Mobasher B., Neithalath N. (2019). Fundamental insights into the compressive and flexural response of binder-and aggregate-optimized ultra-high-performance concrete (UHPC). Cem. Concr. Compos..

[18] Chaabene W.B., Flah M., Nehdi M.L. (2020). Machine learning prediction of mechanical properties of concrete: Critical review. Constr. Build. Mater..

[19] Behnood A., Golafshani E.M. (2020). Machine learning study of the mechanical properties of concretes containing waste foundry sand. Constr. Build. Mater..

[20] Han T., Siddique A., Khayat K., Huang J., Kumar A. (2020). An ensemble machine learning approach for prediction and optimization of modulus of elasticity of recycled aggregate concrete. Constr. Build. Mater..

[21] Zhang J., Huang Y., Aslani F., Ma G., Nener B. (2020). A hybrid intelligent system for designing optimal proportions of recycled aggregate concrete. J. Clean. Prod..

[22] Deng F., He Y., Zhou S., Yu Y., Cheng H., Wu X. (2018). Compressive strength prediction of recycled concrete based on deep learning. Constr. Build. Mater..

[23] Castelli M., Vanneschi L., Silva S. (2013). Prediction of high-performance concrete strength using genetic programming with geometric semantic genetic operators. Expert Syst. Appl..

[24] Han Q., Gui C., Xu J., Lacidogna G. (2019). A generalized method to predict the compressive strength of high-performance concrete by improved random forest algorithm. Constr. Build. Mater..

[25] Al-Shamiri A.K., Yuan T.-F. (2020). Non-tuned machine learning approach for predicting the compressive strength of high-performance concrete. Materials.

[26] Dingqiang F., Rui Y., Zhonghe S., Chunfeng W., Jinnan W., Qiqi S. (2020). A novel approach for developing a green Ultra-High-Performance Concrete (UHPC) with advanced particles packing meso-structure. Constr. Build. Mater..

[27] Fan D., Yu R., Shui Z., Wu C., Song Q., Liu Z., Sun Y., Gao X., He Y. (2020). A new design approach of steel fibre reinforced ultra-high-performance concrete composites: Experiments and modeling. Cem. Concr. Compos..

[28] Marani A., Nehdi M.L. (2020). Machine learning prediction of compressive strength for phase change materials integrated cementitious composites. Constr. Build. Mater..

[29] Suleiman A.R., Nehdi M.L. (2017). Modeling self-healing of concrete using hybrid genetic algorithm–artificial neural network. Materials.

[30] Abuodeh O.R., Abdalla J.A., Hawileh R.A. (2020). Assessment of compressive strength of Ultra-high-Performance Concrete using deep machine learning techniques. Appl. Soft Comput..

[31] Yoo D.-Y., Shin H.-O., Yang J.-M., Yoon Y.-S. (2014). Material and bond properties of ultra-high-performance fiber reinforced concrete with micro steel fibers. Compos. Part B Eng..

[32] Yu R., Spiesz P., Brouwers H. (2015). Development of Ultra-High Performance Fibre Reinforced Concrete (UHPFRC): Towards an efficient utilization of binders and fibres. Constr. Build. Mater..

[33] Wille K., Boisvert-Cotulio C. (2015). Material efficiency in the design of ultra-high-performance concrete. Constr. Build. Mater..

[34] Wu Z., Shi C., He W., Wang D. (2017). Static and dynamic compressive properties of ultra-high-performance concrete (UHPC) with hybrid steel fiber reinforcements. Cem. Concr. Compos..

[35] Song Q., Yu R., Shui Z., Wang X., Rao S., Lin Z. (2018). Optimization of fibre orientation and distribution for a sustainable Ultra-High Performance Fibre Reinforced Concrete (UHPFRC): Experiments and mechanism analysis. Constr. Build. Mater..

[36] Kang S.-H., Jeong Y., Tan K.H., Moon J. (2018). The use of limestone to replace physical filler of quartz powder in UHPFRC. Cem. Concr. Compos..

[37] Rajasekar A., Arunachalam K., Kottaisamy M. (2019). Assessment of strength and durability characteristics of copper slag incorporated ultra-high strength concrete. J. Clean. Prod..

[38] Yoo D.-Y., Kim M.-J. (2019). High energy absorbent ultra-high-performance concrete with hybrid steel and polyethylene fibers. Constr. Build. Mater..

[39] Li Y., Tan K.H., Yang E.-H. (2019). Synergistic effects of hybrid polypropylene and steel fibers on explosive spalling prevention of ultra-high-performance concrete at elevated temperature. Cem. Concr. Compos..

[40] Kang S.-H., Hong S.-G., Moon J. (2019). The use of rice husk ash as reactive filler in ultra-high-performance concrete. Cem. Concr. Res..

[41] Ghafari E., Costa H., Júlio E., Portugal A., Durães L. (2014). The effect of nanosilica addition on flowability, strength and transport properties of ultra-high-performance concrete. Mater. Design.

[42] Gesoglu M., Güneyisi E., Asaad D.S., Muhyaddin G.F. (2016). Properties of low binder ultra-high-performance cementitious composites: Comparison of nanosilica and microsilica. Constr. Build. Mater..

[43] Khaloo A., Mobini M.H., Hosseini P. (2016). Influence of different types of nano-SiO2 particles on properties of high-performance concrete. Constr. Build. Mater..

[44] Janković K., Stanković S., Bojović D., Stojanović M., Antić L. (2016). The influence of nano-silica and barite aggregate on properties of ultra-high-performance concrete. Constr. Build. Mater..

[45] Ahmad S., Mohaisen K.O., Adekunle S.K., Al-Dulaijan S.U., Maslehuddin M. (2019). Influence of admixing natural pozzolan as partial replacement of cement and microsilica in UHPC mixtures. Constr. Build. Mater..

[46] Zhang H., Ji T., He B., He L. (2019). Performance of ultra-high-performance concrete (UHPC) with cement partially replaced by ground granite powder (GGP) under different curing conditions. Constr. Build. Mater..

[47] Wu Z., Shi C., Khayat K.H., Wan S. (2016). Effects of different nanomaterials on hardening and performance of ultra-high strength concrete (UHSC). Cem. Concr. Compos..

[48] Gesoglu M., Güneyisi E., Muhyaddin G.F., Asaad D.S. (2016). Strain hardening ultra-high-performance fiber reinforced cementitious composites: Effect of fiber type and concentration. Compos. Part B Eng..

[49] Sadrmomtazi A., Tajasosi S., Tahmouresi B. (2018). Effect of materials proportion on rheology and mechanical strength and microstructure of ultra-high-performance concrete (UHPC). Constr. Build. Mater..

[50] Qu D., Cai X., Chang W. (2018). Evaluating the effects of steel fibers on mechanical properties of ultra-high-performance concrete using artificial neural networks. Appl. Sci..

[51] Abellán-García J. (2020). Four-layer perceptron approach for strength prediction of UHPC. Constr. Build. Mater..

[52] Ziolkowski P., Niedostatkiewicz M. (2019). Machine learning techniques in concrete mix design. Materials.

[53] Feng S., Zhou H., Dong H. (2019). Using deep neural network with small dataset to predict material defects. Mater. Des..

[54] Butler K.T., Davies D.W., Cartwright H., Isayev O., Walsh A. (2018). Machine learning for molecular and materials science. Nature.

[55] Goodfellow I., Pouget-Abadie J., Mirza M., Xu B., Warde-Farley D., Ozair S., Courville A., Bengio Y. Generative adversarial nets. Proceedings of the 27th International Conference on Neural Information Processing Systems.

[56] Fekri M.N., Ghosh A.M., Grolinger K. (2020). Generating energy data for machine learning with recurrent generative adversarial networks. Energies.

[57] Xu L., Veeramachaneni K. (2018). Synthesizing tabular data using generative adversarial networks. arXiv.

[58] Mirza M., Osindero S. (2014). Conditional generative adversarial nets. arXiv.

[59] Arjovsky M., Chintala S., Bottou L. (2017). Wasserstein gan. arXiv.

[60] Zhu J.-Y., Park T., Isola P., Efros A.A. Unpaired image-to-image translation using cycle-consistent adversarial networks. Proceedings of the 2017 IEEE International Conference on Computer Vision.

[61] Breiman L., Friedman J., Stone C.J., Olshen R.A. (1984). Classification and Regression Trees.

[62] Ahmad M.W., Mourshed M., Rezgui Y. (2018). Tree-based ensemble methods for predicting PV power generation and their comparison with support vector regression. Energy.

[63] Breiman L. (2001). Random forests. Mach. Learn..

[64] Pedregosa F., Varoquaux G., Gramfort A., Michel V., Thirion B., Grisel O., Blondel M., Prettenhofer P., Weiss R., Dubourg V. (2011). Scikit-learn: Machine learning in Python. J. Mach. Learn. Res..

[65] Ahmad M.W., Reynolds J., Rezgui Y. (2018). Predictive modelling for solar thermal energy systems: A comparison of support vector regression, random forest, extra trees and regression trees. J. Clean. Prod..

[66] Geurts P., Ernst D., Wehenkel L. (2006). Extremely randomized trees. Mach. Learn..

[67] Friedman J.H. (2002). Stochastic gradient boosting. Comput. Stat. Data Anal..

[68] Ke G., Meng Q., Finley T., Wang T., Chen W., Ma W., Ye Q., Liu T.-Y. Lightgbm: A highly efficient gradient boosting decision tree. Proceedings of the Neural Information Processing Systems 2017.

[69] Persson C., Bacher P., Shiga T., Madsen H. (2017). Multi-site solar power forecasting using gradient boosted regression trees. Sol. Energy.

[70] El Kababji S., Srikantha P. (2020). A Data-Driven Approach for Generating Synthetic Load Patterns and Usage Habits. IEEE Trans. Smart Grid.

[71] Esteban C., Hyland S.L., Rätsch G. (2017). Real-valued (medical) time series generation with recurrent conditional gans. arXiv.

[72] Browne M.W. (2000). Cross-validation methods. J. Math. Psychol..

[73] Sih G.C., Ditomasso A. (2012). Fracture Mechanics of Concrete: Structural Application and Numerical Calculation: Structural Application and Numerical Calculation.

[74] Kumar S., Barai S.V. (2011). Introduction to Fracture Mechanics of Concrete. Concrete Fracture Models and Applications.

[75] Kurumatani M., Terada K., Kato J., Kyoya T., Kashiyama K. (2016). An isotropic damage model based on fracture mechanics for concrete. Eng. Fract. Mech..

[76] Schlangen E., Van Mier J. (1992). Simple lattice model for numerical simulation of fracture of concrete materials and structures. Mater. Struct..

[77] Lilliu G., van Mier J.G. (2003). 3D lattice type fracture model for concrete. Eng. Fract. Mech..

[78] Smith J., Cusatis G., Pelessone D., Landis E., O’Daniel J., Baylot J. (2014). Discrete modeling of ultra-high-performance concrete with application to projectile penetration. Int. J. Impact Eng..

[79] Pan Z., Ma R., Wang D., Chen A. (2018). A review of lattice type model in fracture mechanics: Theory, applications, and perspectives. Eng. Fract. Mech..

[80] Eftekhari M., Ardakani S.H., Mohammadi S. (2014). An XFEM multiscale approach for fracture analysis of carbon nanotube reinforced concrete. Theor. Appl. Fract. Mech..

[81] Schlangen E., Garboczi E.J. (1997). Fracture simulations of concrete using lattice models: Computational aspects. Eng. Fract. Mech..

[82] Ngo T., Mendis P., Krauthammer T. (2007). Behavior of ultrahigh strength prestressed concrete panels subjected to blast loading. J. Struct. Eng..

[83] Hwang Y.K., Bolander J.E., Lim Y.M. (2020). Evaluation of dynamic tensile strength of concrete using lattice-based simulations of spalling tests. Int. J. Fract..

