# Supplementary material for: Predicting Ultra-High-Performance Concrete Compressive Strength Using Tabular Generative Adversarial Networks
Source: Materials (Basel). 2020 Oct 24;13(21):4757. doi: 10.3390/ma13214757 (PMC7663629; doi:10.3390/ma13214757)
Supplement: Supplementary file 1 [file materials-13-04757-s001.pdf]

materials

MDPI

Supplementary Materials

Predicting Ultra-High-Performance Concrete Compressive Strength Using Tabular Generative Adversarial Networks

Afshin Marani [1], Armin Jamali [2] and Moncef L. Nehdi [1,*]

[1] Department of Civil and Environmental Engineering, Western University, London, Ontario, Canada, N6A 5B9; amarani@uwo.ca
[2] Department of Civil Engineering, K.N. Toosi University of Technology, Tehran, Iran, 1969764499; armin.jamali69@gmail.com
* Correspondence: mnehdi@uwo.ca ; Tel: (519) 661-2111 Ext. 88308; Fax: (51) 661-3779

Received: 22 September 2020; Accepted: 22 October 2020; Published: 24 October 2020

The original UHPC dataset used for developing machine learning models is presented herein. The dataset incorporates 911 data examples of mixture proportions, along with 16 data attributes. **Table S1** presents the input variables of the dataset as well as their designation and units. **Table S2** reports the final dataset used in the original article.

Table S1. Variables considered in final dataset extracted from literature.

Variable	Designation	Unit	Variable	Designation	Unit
Cement	C	kg/m³	Fine aggregate	Sand	kg/m³
Silica fume	SF	kg/m³	Coarse aggregate	Gravel	kg/m³
Slag	S	kg/m³	Fiber	Fi	kg/m³
Fly ash	FA	kg/m³	Superplasticizer	SP	kg/m³
Quartz powder	QP	kg/m³	Temperature	T	°C
Limestone powder	LP	kg/m³	Relative humidity	RH	%
Nano silica	NS	kg/m³	Age	Age	days
Water	W	kg/m³	Compressive strength	f_c'	MPa

Table S2. Final dataset used for machine learning modeling.

Index	C	S	SF	LP	QP	FA	NS	W	Sand	Gravel	Fi	SP	RH	T	Age	f_c'
**0**	450	0	50	0	0	0	0	90	797	1195	0	18.00	95	20	28	131
**1**	630	0	70	0	0	0	0	126	715	1073	0	18.00	95	20	28	135
**2**	810	0	90	0	0	0	0	162	616	923	0	18.00	95	20	28	137
**3**	810	0	90	0	0	0	0	162	616	923	0	18.00	95	20	28	137
**6**	630	180	90	0	0	0	0	126	616	923	0	18.00	95	20	28	150
**7**	630	180	90	0	0	0	0	144	616	923	0	18.00	95	20	28	142
**9**	630	180	90	0	0	0	0	144	616	923	0	18.00	95	20	28	142
**10**	450	180	90	180	0	0	0	144	616	923	0	18.00	95	20	28	150
**11**	270	180	90	360	0	0	0	144	616	923	0	18.00	95	20	28	130
**12**	450	0	50	0	0	0	0	90	797	1195	0	18.00	95	20	56	139
**13**	630	0	70	0	0	0	0	126	715	1073	0	18.00	95	20	56	146
**14**	810	0	90	0	0	0	0	162	616	923	0	18.00	95	20	56	150
**15**	810	0	90	0	0	0	0	162	616	923	0	18.00	95	20	56	150
**16**	630	180	90	0	0	0	0	162	616	923	0	18.00	95	20	56	141
**17**	450	360	90	0	0	0	0	162	616	923	0	18.00	95	20	56	133
**18**	630	180	90	0	0	0	0	126	616	923	0	18.00	95	20	56	159
**19**	630	180	90	0	0	0	0	144	616	923	0	18.00	95	20	56	160
**20**	630	180	90	0	0	0	0	162	616	923	0	18.00	95	20	56	141
**21**	630	180	90	0	0	0	0	144	616	923	0	18.00	95	20	56	160
**22**	450	180	90	180	0	0	0	144	616	923	0	18.00	95	20	56	161

**23**	270	180	90	360	0	0	0	144	616	923	0	18.00	95	20	56	139
**24**	450	0	50	0	0	0	0	90	797	1195	0	18.00	95	20	90	151
**25**	630	0	70	0	0	0	0	126	715	1073	0	18.00	95	20	90	160
**26**	810	0	90	0	0	0	0	162	616	923	0	18.00	95	20	90	170
**27**	810	0	90	0	0	0	0	162	616	923	0	18.00	95	20	90	170
**28**	630	180	90	0	0	0	0	162	616	923	0	18.00	95	20	90	169
**30**	630	180	90	0	0	0	0	126	616	923	0	18.00	95	20	90	168
**31**	630	180	90	0	0	0	0	144	616	923	0	18.00	95	20	90	172
**32**	630	180	90	0	0	0	0	162	616	923	0	18.00	95	20	90	169
**33**	630	180	90	0	0	0	0	144	616	923	0	18.00	95	20	90	172
**34**	450	180	90	180	0	0	0	144	616	923	0	18.00	95	20	90	176
**35**	270	180	90	360	0	0	0	144	616	923	0	18.00	95	20	90	150
**36**	450	0	50	0	0	0	0	90	797	1195	0	18.00	95	20	180	159
**37**	630	0	70	0	0	0	0	126	715	1073	0	18.00	95	20	180	167
**38**	810	0	90	0	0	0	0	162	616	923	0	18.00	95	20	180	174
**39**	810	0	90	0	0	0	0	162	616	923	0	18.00	95	20	180	174
**40**	630	180	90	0	0	0	0	162	616	923	0	18.00	95	20	180	170
**42**	630	180	90	0	0	0	0	126	616	923	0	18.00	95	20	180	168
**43**	630	180	90	0	0	0	0	144	616	923	0	18.00	95	20	180	175
**44**	630	180	90	0	0	0	0	162	616	923	0	18.00	95	20	180	170
**45**	630	180	90	0	0	0	0	144	616	923	0	18.00	95	20	180	175
**46**	450	180	90	180	0	0	0	144	616	923	0	18.00	95	20	180	179
**47**	270	180	90	360	0	0	0	144	616	923	0	18.00	95	20	180	160
**48**	450	0	50	0	0	0	0	90	797	1195	0	18.00	95	20	365	161
**49**	630	0	70	0	0	0	0	126	715	1073	0	18.00	95	20	365	169
**50**	810	0	90	0	0	0	0	162	616	923	0	18.00	95	20	365	177
**51**	810	0	90	0	0	0	0	162	616	923	0	18.00	95	20	365	177
**52**	630	180	90	0	0	0	0	162	616	923	0	18.00	95	20	365	174
**54**	630	180	90	0	0	0	0	126	616	923	0	18.00	95	20	365	168
**55**	630	180	90	0	0	0	0	144	616	923	0	18.00	95	20	365	178
**56**	630	180	90	0	0	0	0	162	616	923	0	18.00	95	20	365	174
**57**	630	180	90	0	0	0	0	144	616	923	0	18.00	95	20	365	178
**58**	450	180	90	180	0	0	0	144	616	923	0	18.00	95	20	365	183
**59**	270	180	90	360	0	0	0	144	616	923	0	18.00	95	20	365	164
**60**	874.9	0	43.7	0	0	0	0	202.1	1273.4	0	0	45.90	100	21	7	85.91
**61**	612.4	0	43.7	262.5	0	0	0	202.1	1273.4	0	0	45.90	100	21	7	78.52
**62**	699.9	0	43.7	0	175	0	0	202.1	1273.4	0	0	45.90	100	21	7	85.91
**63**	874.9	0	43.7	0	0	0	0	202.1	1273.4	0	39	45.90	100	21	7	106.71
**64**	612.4	0	43.7	262.5	0	0	0	202.1	1273.4	0	39	45.90	100	21	7	89.93
**65**	699.9	0	43.7	0	175	0	0	202.1	1273.4	0	39	45.90	100	21	7	99.33
**66**	874.9	0	43.7	0	0	0	0	202.1	1273.4	0	78	45.90	100	21	7	108.73
**67**	612.4	0	43.7	262.5	0	0	0	202.1	1273.4	0	78	45.90	100	21	7	92.62
**68**	699.9	0	43.7	0	175	0	0	202.1	1273.4	0	78	45.90	100	21	7	102.01
**69**	874.9	0	43.7	0	0	0	0	202.1	1273.4	0	117	45.90	100	21	7	110.74
**70**	612.4	0	43.7	262.5	0	0	0	202.1	1273.4	0	117	45.90	100	21	7	100
**71**	699.9	0	43.7	0	175	0	0	202.1	1273.4	0	117	45.90	100	21	7	108.73
**72**	874.9	0	43.7	0	0	0	0	202.1	1273.4	0	156	45.90	100	21	7	120.81
**73**	612.4	0	43.7	262.5	0	0	0	202.1	1273.4	0	156	45.90	100	21	7	105.37
**74**	699.9	0	43.7	0	175	0	0	202.1	1273.4	0	156	45.90	100	21	7	114.77
**76**	612.4	0	43.7	262.5	0	0	0	202.1	1273.4	0	195	45.90	100	21	7	114.09
**77**	699.9	0	43.7	0	175	0	0	202.1	1273.4	0	195	45.90	100	21	7	122.82
**78**	874.9	0	43.7	0	0	0	0	202.1	1273.4	0	0	45.90	100	21	28	100.67
**79**	612.4	0	43.7	262.5	0	0	0	202.1	1273.4	0	0	45.90	100	21	28	88.59
**81**	874.9	0	43.7	0	0	0	0	202.1	1273.4	0	39	45.90	100	21	28	115.44
**82**	612.4	0	43.7	262.5	0	0	0	202.1	1273.4	0	39	45.90	100	21	28	100.67
**83**	699.9	0	43.7	0	175	0	0	202.1	1273.4	0	39	45.90	100	21	28	106.71
**84**	874.9	0	43.7	0	0	0	0	202.1	1273.4	0	78	45.90	100	21	28	124.83
**85**	612.4	0	43.7	262.5	0	0	0	202.1	1273.4	0	78	45.90	100	21	28	114.09
**86**	699.9	0	43.7	0	175	0	0	202.1	1273.4	0	78	45.90	100	21	28	120.81
**87**	874.9	0	43.7	0	0	0	0	202.1	1273.4	0	117	45.90	100	21	28	132.22
**88**	612.4	0	43.7	262.5	0	0	0	202.1	1273.4	0	117	45.90	100	21	28	126.17
**89**	699.9	0	43.7	0	175	0	0	202.1	1273.4	0	117	45.90	100	21	28	130.2
**90**	874.9	0	43.7	0	0	0	0	202.1	1273.4	0	156	45.90	100	21	28	146.31

**91**	612.4	0	43.7	262.5	0	0	0	202.1	1273.4	0	156	45.90	100	21	28	134.23
**92**	699.9	0	43.7	0	175	0	0	202.1	1273.4	0	156	45.90	100	21	28	139.6
**93**	874.9	0	43.7	0	0	0	0	202.1	1273.4	0	195	45.90	100	21	28	155.71
**94**	612.4	0	43.7	262.5	0	0	0	202.1	1273.4	0	195	45.90	100	21	28	141.61
**95**	699.9	0	43.7	0	175	0	0	202.1	1273.4	0	195	45.90	100	21	28	148.32
**96**	729	0	124	0	397	0	0	170	833	0	156	30.00	100	23	28	166.1
**97**	401	0	124	0	397	328	0	170	833	0	156	30.00	100	23	28	124.7
**98**	401	328	124	0	397	0	0	170	833	0	156	30.00	100	23	28	139.4
**100**	439.5	0	0	263.7	175.9	0	0	175.8	1273.4	0	0	43.90	100	21	3	49.61
**101**	435.1	0	0	263.7	175.9	0	4.4	175.8	1273.4	0	0	43.90	100	21	3	50.87
**102**	430.7	0	0	263.7	175.9	0	8.8	175.8	1273.4	0	0	43.90	100	21	3	54.96
**103**	426.3	0	0	263.7	175.9	0	13.2	175.8	1273.4	0	0	43.90	100	21	3	56.85
**104**	421.9	0	0	263.7	175.9	0	17.6	175.8	1273.4	0	0	43.90	100	21	3	57.17
**105**	417.5	0	0	263.7	175.9	0	22	175.8	1273.4	0	0	43.90	100	21	3	55.91
**107**	435.1	0	0	263.7	175.9	0	4.4	175.8	1273.4	0	195	43.90	100	21	3	70.35
**108**	430.7	0	0	263.7	175.9	0	8.8	175.8	1273.4	0	195	43.90	100	21	3	72.84
**109**	426.3	0	0	263.7	175.9	0	13.2	175.8	1273.4	0	195	43.90	100	21	3	78.44
**110**	421.9	0	0	263.7	175.9	0	17.6	175.8	1273.4	0	195	43.90	100	21	3	79.69
**111**	417.5	0	0	263.7	175.9	0	22	175.8	1273.4	0	195	43.90	100	21	3	77.82
**114**	430.7	0	0	263.7	175.9	0	8.8	175.8	1273.4	0	0	43.90	100	21	7	65.98
**115**	426.3	0	0	263.7	175.9	0	13.2	175.8	1273.4	0	0	43.90	100	21	7	68.82
**116**	421.9	0	0	263.7	175.9	0	17.6	175.8	1273.4	0	0	43.90	100	21	7	69.13
**117**	417.5	0	0	263.7	175.9	0	22	175.8	1273.4	0	0	43.90	100	21	7	65.35
**118**	439.5	0	0	263.7	175.9	0	0	175.8	1273.4	0	195	43.90	100	21	7	90.89
**119**	435.1	0	0	263.7	175.9	0	4.4	175.8	1273.4	0	195	43.90	100	21	7	94.63
**120**	430.7	0	0	263.7	175.9	0	8.8	175.8	1273.4	0	195	43.90	100	21	7	96.5
**121**	426.3	0	0	263.7	175.9	0	13.2	175.8	1273.4	0	195	43.90	100	21	7	100.86
**122**	421.9	0	0	263.7	175.9	0	17.6	175.8	1273.4	0	195	43.90	100	21	7	103.35
**123**	417.5	0	0	263.7	175.9	0	22	175.8	1273.4	0	195	43.90	100	21	7	98.99
**125**	435.1	0	0	263.7	175.9	0	4.4	175.8	1273.4	0	0	43.90	100	21	28	77.01
**126**	430.7	0	0	263.7	175.9	0	8.8	175.8	1273.4	0	0	43.90	100	21	28	81.1
**127**	426.3	0	0	263.7	175.9	0	13.2	175.8	1273.4	0	0	43.90	100	21	28	88.66
**128**	421.9	0	0	263.7	175.9	0	17.6	175.8	1273.4	0	0	43.90	100	21	28	91.18
**129**	417.5	0	0	263.7	175.9	0	22	175.8	1273.4	0	0	43.90	100	21	28	86.46
**130**	439.5	0	0	263.7	175.9	0	0	175.8	1273.4	0	195	43.90	100	21	28	113.93
**131**	435.1	0	0	263.7	175.9	0	4.4	175.8	1273.4	0	195	43.90	100	21	28	120.16
**132**	430.7	0	0	263.7	175.9	0	8.8	175.8	1273.4	0	195	43.90	100	21	28	126.38
**136**	594.2	0	0	265.3	0	0	24.8	176.9	1282.3	0	0	44.20	100	21	28	98.89
**137**	594.2	0	0	265.3	0	0	24.8	176.9	1282.3	0	156	44.20	100	21	28	139.44
**138**	594.2	0	0	265.3	0	0	24.8	176.9	1282.3	0	156	44.20	100	21	28	141.67
**139**	594.2	0	0	265.3	0	0	24.8	176.9	1282.3	0	156	44.20	100	21	28	129.44
**140**	594.2	0	0	265.3	0	0	24.8	176.9	1282.3	0	156	44.20	100	21	28	124.44
**141**	594.2	0	0	265.3	0	0	24.8	176.9	1282.3	0	156	44.20	100	21	28	120.56
**142**	594.2	0	0	265.3	0	0	24.8	176.9	1282.3	0	156	44.20	100	21	28	129.01
**143**	594.2	0	0	265.3	0	0	24.8	176.9	1282.3	0	156	44.20	100	21	28	132.96
**144**	594.2	0	0	265.3	0	0	24.8	176.9	1282.3	0	156	44.20	100	21	28	129.58
**145**	594.2	0	0	265.3	0	0	24.8	176.9	1282.3	0	156	44.20	100	21	28	136.34
**146**	594.2	0	0	265.3	0	0	24.8	176.9	1282.3	0	0	44.20	100	21	7	86.11
**147**	594.2	0	0	265.3	0	0	24.8	176.9	1282.3	0	156	44.20	100	21	7	113.89
**148**	594.2	0	0	265.3	0	0	24.8	176.9	1282.3	0	156	44.20	100	21	7	117.22
**149**	594.2	0	0	265.3	0	0	24.8	176.9	1282.3	0	156	44.20	100	21	7	114.44
**150**	594.2	0	0	265.3	0	0	24.8	176.9	1282.3	0	156	44.20	100	21	7	112.22
**151**	594.2	0	0	265.3	0	0	24.8	176.9	1282.3	0	156	44.20	100	21	7	111.11
**153**	753	0	188	0	0	183	0	180.72	1129	0	0	27.00	100	20	28	166
**154**	745	0	186	0	0	181	0	171.35	1118	0	0	27.00	100	20	28	162
**156**	778	0	194	0	0	189	0	178.94	0	1167	0	28.00	100	20	28	181
**157**	758	0	190	0	0	184	0	166.76	0	1138	0	27.00	100	20	28	155
**158**	745	0	186	0	0	181	0	171.35	0	1118	0	27.00	100	20	28	159
**159**	890	0	0	0	0	0	0	178	1231	0	0	30.20	100	21	1	59
**160**	890	0	0	0	0	0	0	178	1231	0	0	30.20	100	21	1	70.7
**161**	845.5	0	44.5	0	0	0	0	178	1231	0	0	30.20	100	21	1	73.2
**162**	801	0	89	0	0	0	0	178	1231	0	0	30.20	100	21	1	75.7
**163**	756.5	0	133.5	0	0	0	0	178	1231	0	0	30.20	100	21	1	70.5

**164**	712	0	178	0	0	0	0	178	1231	0	0	30.20	100	21	1	62.1
**165**	845.5	0	44.5	0	0	0	0	178	1231	0	0	30.20	100	21	1	77.8
**166**	712	0	0	0	0	178	0	178	1231	0	0	30.20	100	21	1	53.7
**169**	667.5	0	44.5	0	0	178	0	178	1231	0	0	30.20	100	21	1	52.8
**170**	1009	0	0	0	0	0	0	201.8	1231	0	0	34.20	100	21	1	72.8
**171**	1009	0	0	0	0	0	0	201.8	1231	0	0	34.20	100	21	1	73.2
**172**	958.55	0	50.45	0	0	0	0	201.8	1231	0	0	34.20	100	21	1	80.1
**173**	756.75	0	50.45	0	0	201.8	0	201.8	1231	0	0	34.20	100	21	1	53.1
**174**	890	0	0	0	0	0	0	178	1231	0	0	30.20	100	21	7	95.7
**175**	890	0	0	0	0	0	0	178	1231	0	0	30.20	100	21	7	97.4
**176**	845.5	0	44.5	0	0	0	0	178	1231	0	0	30.20	100	21	7	105.7
**177**	801	0	89	0	0	0	0	178	1231	0	0	30.20	100	21	7	102.6
**178**	756.5	0	133.5	0	0	0	0	178	1231	0	0	30.20	100	21	7	96.8
**179**	712	0	178	0	0	0	0	178	1231	0	0	30.20	100	21	7	95.9
**180**	845.5	0	44.5	0	0	0	0	178	1231	0	0	30.20	100	21	7	106.1
**181**	712	0	0	0	0	178	0	178	1231	0	0	30.20	100	21	7	99.2
**182**	623	0	0	0	0	267	0	178	1231	0	0	30.20	100	21	7	101.2
**183**	534	0	0	0	0	356	0	178	1231	0	0	30.20	100	21	7	75.8
**184**	667.5	0	44.5	0	0	178	0	178	1231	0	0	30.20	100	21	7	92.8
**185**	1009	0	0	0	0	0	0	201.8	1231	0	0	34.20	100	21	7	102.8
**186**	1009	0	0	0	0	0	0	201.8	1231	0	0	34.20	100	21	7	102.3
**187**	958.55	0	50.45	0	0	0	0	201.8	1231	0	0	34.20	100	21	7	102.8
**188**	756.75	0	50.45	0	0	201.8	0	201.8	1231	0	0	34.20	100	21	7	101.5
**189**	890	0	0	0	0	0	0	178	1231	0	0	30.20	100	21	28	106.3
**190**	890	0	0	0	0	0	0	178	1231	0	0	30.20	100	21	28	113.2
**191**	845.5	0	44.5	0	0	0	0	178	1231	0	0	30.20	100	21	28	117.2
**192**	801	0	89	0	0	0	0	178	1231	0	0	30.20	100	21	28	118.6
**193**	756.5	0	133.5	0	0	0	0	178	1231	0	0	30.20	100	21	28	118
**194**	712	0	178	0	0	0	0	178	1231	0	0	30.20	100	21	28	111
**195**	845.5	0	44.5	0	0	0	0	178	1231	0	0	30.20	100	21	28	116.6
**196**	712	0	0	0	0	178	0	178	1231	0	0	30.20	100	21	28	109.9
**197**	623	0	0	0	0	267	0	178	1231	0	0	30.20	100	21	28	114.8
**198**	534	0	0	0	0	356	0	178	1231	0	0	30.20	100	21	28	102.6
**199**	667.5	0	44.5	0	0	178	0	178	1231	0	0	30.20	100	21	28	112.8
**200**	1009	0	0	0	0	0	0	201.8	1231	0	0	34.20	100	21	28	113.8
**201**	1009	0	0	0	0	0	0	201.8	1231	0	0	34.20	100	21	28	115.2
**202**	958.55	0	50.45	0	0	0	0	201.8	1231	0	0	34.20	100	21	28	115.4
**203**	756.75	0	50.45	0	0	201.8	0	201.8	1231	0	0	34.20	100	21	28	114.5
**205**	1151.11	0	211.89	0	0	0	0	272.6	1231	0	0	46.20	100	90	28	159.7
**206**	1151.11	0	211.89	0	0	0	0	272.6	1231	0	0	46.20	100	90	28	165.8
**207**	1151.11	0	211.89	0	0	0	0	272.6	1231	0	0	46.20	100	90	28	163.9
**209**	1151.11	0	211.89	0	0	0	0	272.6	1231	0	234	46.20	100	90	28	170.6
**210**	1151.11	0	211.89	0	0	0	0	272.6	1231	0	234	46.20	100	90	28	179
**211**	1151.11	0	211.89	0	0	0	0	272.6	1231	0	234	46.20	100	90	28	177
**212**	890	0	0	0	0	0	0	178	1231	0	0	30.20	100	21	56	108.8
**213**	890	0	0	0	0	0	0	178	1231	0	0	30.20	100	21	56	113.8
**214**	845.5	0	44.5	0	0	0	0	178	1231	0	0	30.20	100	21	56	120.1
**215**	801	0	89	0	0	0	0	178	1231	0	0	30.20	100	21	56	127.4
**216**	756.5	0	133.5	0	0	0	0	178	1231	0	0	30.20	100	21	56	120.1
**217**	712	0	178	0	0	0	0	178	1231	0	0	30.20	100	21	56	117.2
**218**	845.5	0	44.5	0	0	0	0	178	1231	0	0	30.20	100	21	56	124.1
**219**	712	0	0	0	0	178	0	178	1231	0	0	30.20	100	21	56	110.3
**220**	623	0	0	0	0	267	0	178	1231	0	0	30.20	100	21	56	117.2
**221**	534	0	0	0	0	356	0	178	1231	0	0	30.20	100	21	56	110.9
**222**	667.5	0	44.5	0	0	178	0	178	1231	0	0	30.20	100	21	56	113.8
**223**	1009	0	0	0	0	0	0	201.8	1231	0	0	34.20	100	21	56	126.2
**224**	1009	0	0	0	0	0	0	201.8	1231	0	0	34.20	100	21	56	129.3
**225**	958.55	0	50.45	0	0	0	0	201.8	1231	0	0	34.20	100	21	56	129
**226**	756.75	0	50.45	0	0	201.8	0	201.8	1231	0	0	34.20	100	21	56	131.7
**227**	890	0	0	0	0	0	0	178	1231	0	0	30.20	100	21	90	114.1
**228**	890	0	0	0	0	0	0	178	1231	0	0	30.20	100	21	90	118.1
**229**	845.5	0	44.5	0	0	0	0	178	1231	0	0	30.20	100	21	90	125.4
**230**	801	0	89	0	0	0	0	178	1231	0	0	30.20	100	21	90	127.6

**231**	756.5	0	133.5	0	0	0	0	178	1231	0	0	30.20	100	21	90	120.9
**232**	712	0	178	0	0	0	0	178	1231	0	0	30.20	100	21	90	118.6
**233**	845.5	0	44.5	0	0	0	0	178	1231	0	0	30.20	100	21	90	126.2
**234**	712	0	0	0	0	178	0	178	1231	0	0	30.20	100	21	90	117.5
**235**	623	0	0	0	0	267	0	178	1231	0	0	30.20	100	21	90	119.3
**236**	534	0	0	0	0	356	0	178	1231	0	0	30.20	100	21	90	119.1
**237**	667.5	0	44.5	0	0	178	0	178	1231	0	0	30.20	100	21	90	119.7
**238**	1009	0	0	0	0	0	0	201.8	1231	0	0	34.20	100	21	90	139.3
**239**	1009	0	0	0	0	0	0	201.8	1231	0	0	34.20	100	21	90	149.7
**240**	958.55	0	50.45	0	0	0	0	201.8	1231	0	0	34.20	100	21	90	155.2
**241**	756.75	0	50.45	0	0	201.8	0	201.8	1231	0	0	34.20	100	21	90	152.1
**242**	472	315	262	0	0	0	0	178	1049	0	0	21.00	100	20	7	94.3
**243**	472	315	262	0	0	0	0	178	1049	0	156	21.00	100	20	7	114.9
**244**	472	315	262	0	0	0	0	178	1049	0	156	21.00	100	20	7	120.9
**245**	472	315	262	0	0	0	0	178	1049	0	156	21.00	100	20	7	108.3
**248**	472	315	262	0	0	0	0	178	1049	0	0	21.00	100	20	28	97.8
**249**	472	315	262	0	0	0	0	178	1049	0	156	21.00	100	20	28	134.4
**250**	472	315	262	0	0	0	0	178	1049	0	156	21.00	100	20	28	143.5
**251**	472	315	262	0	0	0	0	178	1049	0	156	21.00	100	20	28	127.4
**252**	472	315	262	0	0	0	0	178	1049	0	156	21.00	100	20	28	122.9
**253**	472	315	262	0	0	0	0	178	1049	0	156	21.00	100	20	28	120.4
**257**	850	0	137.5	0	0	112.5	0	176	1100	0	234	8.00	100	23	28	162.1
**259**	850	0	137.5	0	0	112.5	0	176	1100	0	234	8.00	100	23	28	162.8
**260**	700	0	50	0	0	150	0	180	1104	0	0	30.00	100	20	1	57.9
**261**	700	0	50	0	0	150	0	180	1104	0	39	30.00	100	20	1	64.5
**262**	700	0	50	0	0	150	0	180	1104	0	78	30.00	100	20	1	73.8
**263**	700	0	50	0	0	150	0	180	1104	0	117	30.00	100	20	1	83.2
**264**	700	0	50	0	0	150	0	180	1104	0	156	30.00	100	20	1	88.8
**265**	700	0	50	0	0	150	0	180	1104	0	195	30.00	100	20	1	89.7
**266**	700	0	50	0	0	150	0	180	1104	0	0	30.00	100	20	7	94.4
**267**	700	0	50	0	0	150	0	180	1104	0	39	30.00	100	20	7	96.3
**268**	700	0	50	0	0	150	0	180	1104	0	78	30.00	100	20	7	102.8
**269**	700	0	50	0	0	150	0	180	1104	0	117	30.00	100	20	7	115.9
**270**	700	0	50	0	0	150	0	180	1104	0	156	30.00	100	20	7	120.6
**271**	700	0	50	0	0	150	0	180	1104	0	195	30.00	100	20	7	125.2
**272**	700	0	50	0	0	150	0	180	1104	0	0	30.00	100	20	28	105.6
**274**	700	0	50	0	0	150	0	180	1104	0	78	30.00	100	20	28	138.3
**275**	700	0	50	0	0	150	0	180	1104	0	117	30.00	100	20	28	149.5
**276**	700	0	50	0	0	150	0	180	1104	0	156	30.00	100	20	28	153.3
**277**	700	0	50	0	0	150	0	180	1104	0	195	30.00	100	20	28	156.1
**279**	741	0	185	64.75	194.25	0	0	185	815	0	156	9.00	65	30	1	76
**280**	741	0	185	129.5	129.5	0	0	185	815	0	156	9.00	65	30	1	78
**281**	741	0	185	194.25	64.75	0	0	185	815	0	156	9.00	65	30	1	82
**282**	741	0	185	259	0	0	0	185	815	0	0	9.00	65	30	1	82
**283**	741	0	185	0	259	0	0	185	815	0	156	9.00	65	30	3	117
**284**	741	0	185	64.75	194.25	0	0	185	815	0	156	9.00	65	30	3	118
**285**	741	0	185	129.5	129.5	0	0	185	815	0	156	9.00	65	30	3	111
**286**	741	0	185	194.25	64.75	0	0	185	815	0	156	9.00	65	30	3	116
**287**	741	0	185	259	0	0	0	185	815	0	0	9.00	65	30	3	115
**288**	741	0	185	0	259	0	0	185	815	0	156	9.00	65	30	7	144
**289**	741	0	185	64.75	194.25	0	0	185	815	0	156	9.00	65	30	7	142
**291**	741	0	185	194.25	64.75	0	0	185	815	0	156	9.00	65	30	7	138
**294**	741	0	185	64.75	194.25	0	0	185	815	0	156	9.00	65	30	28	159
**295**	741	0	185	129.5	129.5	0	0	185	815	0	156	9.00	65	30	28	160
**296**	741	0	185	194.25	64.75	0	0	185	815	0	156	9.00	65	30	28	158
**297**	741	0	185	259	0	0	0	185	815	0	0	9.00	65	30	28	155
**298**	741	0	185	0	259	0	0	185	815	0	156	9.00	65	30	90	165
**299**	741	0	185	64.75	194.25	0	0	185	815	0	156	9.00	65	30	90	171
**300**	741	0	185	129.5	129.5	0	0	185	815	0	156	9.00	65	30	90	167
**301**	741	0	185	194.25	64.75	0	0	185	815	0	156	9.00	65	30	90	168
**305**	890	0	222	0	0	0	0	222	799	0	117	29.70	100	23	28	130.3
**306**	933	0	233	0	0	0	0	168	1026	0	0	37.60	100	23	28	101.4
**307**	933	0	233	0	0	0	0	168	1026	0	0	37.60	100	23	28	116.2

**310**	850	0	260	0	212	0	0	170	850	0	156	45.00	100	23	28	170.4
**311**	850	0	260	0	212	0	0	170	850	0	156	45.00	100	23	28	184
**312**	950	0	234	0	95	0	0	215	1030	0	234	12.70	100	23	28	158.1
**313**	950	0	234	0	95	0	0	215	1030	0	234	12.70	100	23	28	168.6
**315**	850	0	260	0	212	0	0	170	850	0	156	45.00	100	23	28	180.8
**316**	850	0	260	0	212	0	0	170	850	0	156	45.00	100	23	28	192.2
**317**	850	0	260	0	212	0	0	170	850	0	156	45.00	100	23	28	206.6
**318**	850	0	260	0	212	0	0	170	850	0	156	45.00	100	23	28	185.2
**319**	850	0	260	0	212	0	0	170	850	0	156	45.00	100	23	28	198.5
**321**	850	0	260	0	212	0	0	170	850	0	156	45.00	100	23	28	188.2
**322**	850	0	260	0	212	0	0	170	850	0	156	45.00	100	23	28	202.2
**324**	850	0	260	0	212	0	0	170	850	0	156	45.00	100	23	28	193.5
**325**	850	0	260	0	212	0	0	170	850	0	156	45.00	100	23	28	203.9
**327**	850	0	260	0	212	0	0	170	850	0	156	45.00	100	23	28	197.9
**330**	850	0	260	0	212	0	0	170	850	0	156	45.00	100	23	28	201.2
**333**	850	0	260	0	212	0	0	170	850	0	156	45.00	100	23	28	206.2
**336**	850	0	260	0	212	0	0	170	850	0	156	45.00	100	23	28	205.6
**339**	850	0	260	0	212	0	0	170	850	0	156	45.00	100	23	28	199.9
**342**	850	0	260	0	212	0	0	170	850	0	156	45.00	100	23	28	195.2
**345**	850	0	260	0	212	0	0	170	850	0	156	45.00	100	23	28	190.2
**346**	850	0	260	0	212	0	0	170	850	0	156	45.00	100	23	28	208.2
**348**	850	0	260	0	212	0	0	170	850	0	156	45.00	100	23	28	181
**349**	850	0	260	0	212	0	0	170	850	0	156	45.00	100	23	28	192.2
**350**	850	0	260	0	212	0	0	170	850	0	156	45.00	100	23	28	206.78
**351**	850	0	260	0	212	0	0	170	850	0	156	45.00	100	23	28	182.4
**352**	850	0	260	0	212	0	0	170	850	0	156	45.00	100	23	28	194.9
**353**	850	0	260	0	212	0	0	170	850	0	156	45.00	100	23	28	207.8
**354**	850	0	260	0	212	0	0	170	850	0	156	45.00	100	23	28	186.1
**355**	850	0	260	0	212	0	0	170	850	0	156	45.00	100	23	28	196.9
**357**	850	0	260	0	212	0	0	170	850	0	156	45.00	100	23	28	186.8
**358**	850	0	260	0	212	0	0	170	850	0	156	45.00	100	23	28	197.6
**360**	850	0	260	0	212	0	0	170	850	0	156	45.00	100	23	28	187.5
**361**	850	0	260	0	212	0	0	170	850	0	156	45.00	100	23	28	200
**363**	850	0	260	0	212	0	0	170	850	0	156	45.00	100	23	28	173.2
**364**	850	0	260	0	212	0	0	170	850	0	156	45.00	100	23	28	184.4
**365**	850	0	260	0	212	0	0	170	850	0	156	45.00	100	23	28	196.9
**367**	850	0	260	0	212	0	0	170	850	0	156	45.00	100	23	28	181
**368**	850	0	260	0	212	0	0	170	850	0	156	45.00	100	23	28	191.2
**370**	850	0	260	0	212	0	0	170	850	0	156	45.00	100	23	28	176.6
**371**	850	0	260	0	212	0	0	170	850	0	156	45.00	100	23	28	182.4
**373**	850	0	260	0	212	0	0	170	850	0	156	45.00	100	23	28	172.9
**374**	850	0	260	0	212	0	0	170	850	0	156	45.00	100	23	28	181.7
**376**	850	0	260	0	212	0	0	170	850	0	156	45.00	100	23	28	171.2
**377**	850	0	260	0	212	0	0	170	850	0	156	45.00	100	23	28	180.3
**380**	850	0	260	0	212	0	0	170	850	0	156	45.00	100	23	28	174.6
**381**	788.5	0	197.1	0	0	0	0	160.3	1104	0	156	50.00	50	23	28	187.1
**382**	788.5	0	197.1	0	0	0	0	160.3	1104	0	156	50.00	50	23	28	192.3
**383**	788.5	0	197.1	0	0	0	0	160.3	1104	0	156	50.00	50	23	28	204.4
**384**	833	0	208	0	0	0	0	208	1125	0	0	25.00	100	23	28	147
**385**	825	0	206	0	0	0	0	206	1114	0	78	25.00	100	23	28	145.5
**386**	817	0	204	0	0	0	0	204	1102	0	156	25.00	100	23	28	147.5
**387**	808	0	202	0	0	0	0	202	1091	0	234	24.00	100	23	28	165.7
**388**	800	0	200	0	200	0	0	200	880	0	0	9.60	60	20	7	101.5
**389**	800	0	100	0	200	0	0	200	980	0	0	9.60	60	20	7	111.4
**390**	800	0	200	0	100	0	0	200	980	0	0	9.60	60	20	7	108.6
**391**	800	0	200	0	0	0	0	200	1080	0	0	14.40	60	20	7	111
**394**	800	0	200	0	200	0	0	200	880	0	0	9.60	60	20	28	141.3
**395**	800	0	100	0	200	0	0	200	980	0	0	9.60	60	20	28	147.3
**396**	800	0	200	0	100	0	0	200	980	0	0	9.60	60	20	28	146.5
**397**	800	0	200	0	0	0	0	200	1080	0	0	14.40	60	20	28	159.2
**400**	800	0	200	0	200	0	0	200	880	0	0	9.60	60	20	90	163.3
**401**	800	0	100	0	200	0	0	200	980	0	0	9.60	60	20	90	166.4
**402**	800	0	200	0	100	0	0	200	980	0	0	9.60	60	20	90	175.9

**404**	800	0	100	0	200	0	0	200	980	0	0	9.60	60	20	90	141.1
**406**	950	0	255	0	0	0	0	189	873	0	0	31.00	100	23	7	104.2
**407**	941.5	0	255	0	0	0	9.5	189	873	0	0	31.00	100	23	7	109.8
**408**	932	0	255	0	0	0	19	189	873	0	0	31.00	100	23	7	112.6
**409**	921.5	0	255	0	0	0	28.5	189	873	0	0	31.00	100	23	7	119.4
**410**	912	0	255	0	0	0	38	189	873	0	0	31.00	100	23	7	114.3
**411**	950	0	255	0	0	0	0	189	873	0	0	31.00	100	23	28	132.5
**412**	941.5	0	255	0	0	0	9.5	189	873	0	0	31.00	100	23	28	135.8
**413**	932	0	255	0	0	0	19	189	873	0	0	31.00	100	23	28	137
**414**	921.5	0	255	0	0	0	28.5	189	873	0	0	31.00	100	23	28	143.2
**415**	912	0	255	0	0	0	38	189	873	0	0	31.00	100	23	28	139.2
**416**	950	0	255	0	0	0	0	189	873	0	0	31.00	100	23	90	138.7
**417**	941.5	0	255	0	0	0	9.5	189	873	0	0	31.00	100	23	90	139.8
**418**	932	0	255	0	0	0	19	189	873	0	0	31.00	100	23	90	141.5
**419**	921.5	0	255	0	0	0	28.5	189	873	0	0	31.00	100	23	90	147.7
**420**	912	0	255	0	0	0	38	189	873	0	0	31.00	100	23	90	140.4
**421**	800	0	0	0	0	0	0	160	1471.3	0	0	21.60	100	23	1	92.2
**422**	796	0	0	0	0	0	4	160	1461.1	0	0	25.20	100	23	1	82.8
**423**	792	0	0	0	0	0	8	160	1450.8	0	0	28.80	100	23	1	78.9
**424**	784	0	0	0	0	0	16	160	1430.2	0	0	36.00	100	23	1	77.9
**425**	776	0	0	0	0	0	24	160	1409.7	0	0	43.20	100	23	1	75.6
**426**	720	0	80	0	0	0	0	160	1422.9	0	0	29.60	100	23	1	81.8
**427**	716	0	80	0	0	0	4	160	1411.6	0	0	33.60	100	23	1	77.8
**428**	712	0	80	0	0	0	8	160	1400.3	0	0	37.60	100	23	1	69.4
**429**	704	0	80	0	0	0	16	160	1379.8	0	0	44.80	100	23	1	61.5
**430**	696	0	80	0	0	0	24	160	1359.2	0	0	52.00	100	23	1	56.7
**431**	800	0	0	0	0	0	0	160	1471.3	0	0	21.60	100	23	3	103.9
**432**	796	0	0	0	0	0	4	160	1461.1	0	0	25.20	100	23	3	100.2
**433**	792	0	0	0	0	0	8	160	1450.8	0	0	28.80	100	23	3	99.3
**434**	784	0	0	0	0	0	16	160	1430.2	0	0	36.00	100	23	3	95.4
**435**	776	0	0	0	0	0	24	160	1409.7	0	0	43.20	100	23	3	94.5
**436**	720	0	80	0	0	0	0	160	1422.9	0	0	29.60	100	23	3	91.2
**437**	716	0	80	0	0	0	4	160	1411.6	0	0	33.60	100	23	3	87.9
**438**	712	0	80	0	0	0	8	160	1400.3	0	0	37.60	100	23	3	86.3
**439**	704	0	80	0	0	0	16	160	1379.8	0	0	44.80	100	23	3	85.3
**440**	696	0	80	0	0	0	24	160	1359.2	0	0	52.00	100	23	3	84.3
**441**	800	0	0	0	0	0	0	160	1471.3	0	0	21.60	100	23	7	108
**442**	796	0	0	0	0	0	4	160	1461.1	0	0	25.20	100	23	7	108.7
**443**	792	0	0	0	0	0	8	160	1450.8	0	0	28.80	100	23	7	109
**444**	784	0	0	0	0	0	16	160	1430.2	0	0	36.00	100	23	7	109.2
**445**	776	0	0	0	0	0	24	160	1409.7	0	0	43.20	100	23	7	106.9
**446**	720	0	80	0	0	0	0	160	1422.9	0	0	29.60	100	23	7	104.3
**447**	716	0	80	0	0	0	4	160	1411.6	0	0	33.60	100	23	7	105.1
**448**	712	0	80	0	0	0	8	160	1400.3	0	0	37.60	100	23	7	106.2
**449**	704	0	80	0	0	0	16	160	1379.8	0	0	44.80	100	23	7	107.1
**450**	696	0	80	0	0	0	24	160	1359.2	0	0	52.00	100	23	7	102.2
**451**	800	0	0	0	0	0	0	160	1471.3	0	0	21.60	100	23	14	112.4
**452**	796	0	0	0	0	0	4	160	1461.1	0	0	25.20	100	23	14	113.1
**453**	792	0	0	0	0	0	8	160	1450.8	0	0	28.80	100	23	14	114.3
**454**	784	0	0	0	0	0	16	160	1430.2	0	0	36.00	100	23	14	115.6
**455**	776	0	0	0	0	0	24	160	1409.7	0	0	43.20	100	23	14	111.5
**456**	720	0	80	0	0	0	0	160	1422.9	0	0	29.60	100	23	14	116.2
**457**	716	0	80	0	0	0	4	160	1411.6	0	0	33.60	100	23	14	118.5
**458**	712	0	80	0	0	0	8	160	1400.3	0	0	37.60	100	23	14	123
**459**	704	0	80	0	0	0	16	160	1379.8	0	0	44.80	100	23	14	126
**460**	696	0	80	0	0	0	24	160	1359.2	0	0	52.00	100	23	14	119.2
**461**	800	0	0	0	0	0	0	160	1471.3	0	0	21.60	100	23	28	115.4
**462**	796	0	0	0	0	0	4	160	1461.1	0	0	25.20	100	23	28	116.8
**463**	792	0	0	0	0	0	8	160	1450.8	0	0	28.80	100	23	28	120.9
**464**	784	0	0	0	0	0	16	160	1430.2	0	0	36.00	100	23	28	123.4
**465**	776	0	0	0	0	0	24	160	1409.7	0	0	43.20	100	23	28	119.1
**466**	720	0	80	0	0	0	0	160	1422.9	0	0	29.60	100	23	28	120.7
**467**	716	0	80	0	0	0	4	160	1411.6	0	0	33.60	100	23	28	125

**468**	712	0	80	0	0	0	8	160	1400.3	0	0	37.60	100	23	28	130.1
**469**	704	0	80	0	0	0	16	160	1379.8	0	0	44.80	100	23	28	131.8
**470**	696	0	80	0	0	0	24	160	1359.2	0	0	52.00	100	23	28	127.3
**471**	800	0	0	0	0	0	0	160	1471.3	0	0	21.60	100	23	56	119.3
**472**	796	0	0	0	0	0	4	160	1461.1	0	0	25.20	100	23	56	120.9
**473**	792	0	0	0	0	0	8	160	1450.8	0	0	28.80	100	23	56	123.4
**474**	784	0	0	0	0	0	16	160	1430.2	0	0	36.00	100	23	56	126
**475**	776	0	0	0	0	0	24	160	1409.7	0	0	43.20	100	23	56	122.3
**476**	720	0	80	0	0	0	0	160	1422.9	0	0	29.60	100	23	56	124.2
**477**	716	0	80	0	0	0	4	160	1411.6	0	0	33.60	100	23	56	128.8
**478**	712	0	80	0	0	0	8	160	1400.3	0	0	37.60	100	23	56	134.8
**479**	704	0	80	0	0	0	16	160	1379.8	0	0	44.80	100	23	56	137.4
**480**	696	0	80	0	0	0	24	160	1359.2	0	0	52.00	100	23	56	131.8
**481**	800	0	0	0	0	0	0	160	1471.3	0	0	21.60	100	23	90	124.1
**482**	796	0	0	0	0	0	4	160	1461.1	0	0	25.20	100	23	90	125.7
**483**	792	0	0	0	0	0	8	160	1450.8	0	0	28.80	100	23	90	130.6
**484**	784	0	0	0	0	0	16	160	1430.2	0	0	36.00	100	23	90	133.3
**485**	776	0	0	0	0	0	24	160	1409.7	0	0	43.20	100	23	90	129.9
**486**	720	0	80	0	0	0	0	160	1422.9	0	0	29.60	100	23	90	130.3
**487**	716	0	80	0	0	0	4	160	1411.6	0	0	33.60	100	23	90	132.8
**488**	712	0	80	0	0	0	8	160	1400.3	0	0	37.60	100	23	90	137.9
**489**	704	0	80	0	0	0	16	160	1379.8	0	0	44.80	100	23	90	141.6
**490**	696	0	80	0	0	0	24	160	1359.2	0	0	52.00	100	23	90	138.3
**491**	550	0	0	0	0	0	0	192.5	678	1017	0	1.10	100	21	3	32.5
**492**	545.875	0	0	0	0	0	4.125	192.5	674	1011	0	4.40	100	21	3	35.3
**493**	541.75	0	0	0	0	0	8.25	192.5	671	1007	0	6.60	100	21	3	39.5
**494**	545.875	0	0	0	0	0	4.125	192.5	674	1011	0	4.40	100	21	3	37.1
**495**	541.75	0	0	0	0	0	8.25	192.5	671	1007	0	6.60	100	21	3	39.1
**496**	550	0	0	0	0	0	0	165	705	1057	0	3.85	100	21	3	45
**497**	545.875	0	0	0	0	0	4.125	165	702	1052	0	6.60	100	21	3	46.4
**498**	541.75	0	0	0	0	0	8.25	165	699	1049	0	8.25	100	21	3	51.6
**499**	545.875	0	0	0	0	0	4.125	165	702	1052	0	6.60	100	21	3	45.4
**500**	541.75	0	0	0	0	0	8.25	165	699	1049	0	8.25	100	21	3	42.2
**501**	550	0	0	0	0	0	0	137.5	732	1098	0	6.60	100	21	3	58.9
**502**	545.875	0	0	0	0	0	4.125	137.5	728	1092	0	9.90	100	21	3	59.8
**503**	541.75	0	0	0	0	0	8.25	137.5	725	1087	0	12.65	100	21	3	63.3
**504**	545.875	0	0	0	0	0	4.125	137.5	728	1092	0	9.90	100	21	3	56.1
**505**	541.75	0	0	0	0	0	8.25	137.5	725	1087	0	12.65	100	21	3	53.1
**506**	550	0	0	0	0	0	0	192.5	678	1017	0	1.10	100	21	7	40.9
**507**	545.875	0	0	0	0	0	4.125	192.5	674	1011	0	4.40	100	21	7	44.9
**508**	541.75	0	0	0	0	0	8.25	192.5	671	1007	0	6.60	100	21	7	51.3
**509**	545.875	0	0	0	0	0	4.125	192.5	674	1011	0	4.40	100	21	7	45.7
**510**	541.75	0	0	0	0	0	8.25	192.5	671	1007	0	6.60	100	21	7	49.3
**511**	550	0	0	0	0	0	0	165	705	1057	0	3.85	100	21	7	57.2
**512**	545.875	0	0	0	0	0	4.125	165	702	1052	0	6.60	100	21	7	59.7
**513**	541.75	0	0	0	0	0	8.25	165	699	1049	0	8.25	100	21	7	60.1
**514**	545.875	0	0	0	0	0	4.125	165	702	1052	0	6.60	100	21	7	52.5
**515**	541.75	0	0	0	0	0	8.25	165	699	1049	0	8.25	100	21	7	50.1
**516**	550	0	0	0	0	0	0	137.5	732	1098	0	6.60	100	21	7	71.6
**517**	545.875	0	0	0	0	0	4.125	137.5	728	1092	0	9.90	100	21	7	76
**518**	541.75	0	0	0	0	0	8.25	137.5	725	1087	0	12.65	100	21	7	75
**519**	545.875	0	0	0	0	0	4.125	137.5	728	1092	0	9.90	100	21	7	69
**520**	541.75	0	0	0	0	0	8.25	137.5	725	1087	0	12.65	100	21	7	67.8
**522**	545.875	0	0	0	0	0	4.125	192.5	674	1011	0	4.40	100	21	28	53.4
**523**	541.75	0	0	0	0	0	8.25	192.5	671	1007	0	6.60	100	21	28	61.8
**524**	545.875	0	0	0	0	0	4.125	192.5	674	1011	0	4.40	100	21	28	54.5
**525**	541.75	0	0	0	0	0	8.25	192.5	671	1007	0	6.60	100	21	28	61
**526**	550	0	0	0	0	0	0	165	705	1057	0	3.85	100	21	28	67.2
**527**	545.875	0	0	0	0	0	4.125	165	702	1052	0	6.60	100	21	28	64.4
**528**	541.75	0	0	0	0	0	8.25	165	699	1049	0	8.25	100	21	28	72.6
**529**	545.875	0	0	0	0	0	4.125	165	702	1052	0	6.60	100	21	28	64.2
**530**	541.75	0	0	0	0	0	8.25	165	699	1049	0	8.25	100	21	28	62.9
**531**	550	0	0	0	0	0	0	137.5	732	1098	0	6.60	100	21	28	85.5

**532**	545.875	0	0	0	0	0	4.125	137.5	728	1092	0	9.90	100	21	28	86.7
**533**	541.75	0	0	0	0	0	8.25	137.5	725	1087	0	12.65	100	21	28	85
**534**	545.875	0	0	0	0	0	4.125	137.5	728	1092	0	9.90	100	21	28	83.6
**535**	541.75	0	0	0	0	0	8.25	137.5	725	1087	0	12.65	100	21	28	84.7
**537**	545.875	0	0	0	0	0	4.125	192.5	674	1011	0	4.40	100	21	90	62
**538**	541.75	0	0	0	0	0	8.25	192.5	671	1007	0	6.60	100	21	90	65.9
**539**	545.875	0	0	0	0	0	4.125	192.5	674	1011	0	4.40	100	21	90	58.4
**540**	541.75	0	0	0	0	0	8.25	192.5	671	1007	0	6.60	100	21	90	65.3
**541**	550	0	0	0	0	0	0	165	705	1057	0	3.85	100	21	90	74.3
**542**	545.875	0	0	0	0	0	4.125	165	702	1052	0	6.60	100	21	90	75.1
**543**	541.75	0	0	0	0	0	8.25	165	699	1049	0	8.25	100	21	90	76.9
**544**	545.875	0	0	0	0	0	4.125	165	702	1052	0	6.60	100	21	90	71.9
**545**	541.75	0	0	0	0	0	8.25	165	699	1049	0	8.25	100	21	90	70.8
**546**	550	0	0	0	0	0	0	137.5	732	1098	0	6.60	100	21	90	93.8
**547**	545.875	0	0	0	0	0	4.125	137.5	728	1092	0	9.90	100	21	90	95
**548**	541.75	0	0	0	0	0	8.25	137.5	725	1087	0	12.65	100	21	90	91.2
**549**	545.875	0	0	0	0	0	4.125	137.5	728	1092	0	9.90	100	21	90	90
**550**	541.75	0	0	0	0	0	8.25	137.5	725	1087	0	12.65	100	21	90	92.6
**551**	950	0	200	0	350	0	0	230	570	0	234	53.00	100	20	28	140.7
**552**	931	0	200	0	350	0	19	230	570	0	234	53.00	100	20	28	150.6
**553**	902.5	0	200	0	350	0	47.5	230	570	0	234	53.00	100	20	28	148.4
**554**	950	0	200	0	350	0	0	230	690	0	234	53.00	100	20	28	144.7
**555**	931	0	200	0	350	0	19	230	690	0	234	53.00	100	20	28	155.4
**556**	902.5	0	200	0	350	0	47.5	230	690	0	234	53.00	100	20	28	142.4
**557**	950	0	200	0	350	0	0	230	810	0	234	53.00	100	20	28	144.5
**559**	902.5	0	200	0	350	0	47.5	230	810	0	234	53.00	100	20	28	137.2
**560**	863	0	216	0	0	0	0	177	923	0	0	32.37	100	20	7	88.9
**561**	647	216	216	0	0	0	0	177	923	0	0	32.37	100	20	7	86.7
**563**	432	216	216	0	0	216	0	177	923	0	0	32.40	100	20	7	79.2
**564**	829	0	216	0	0	0	34.5	177	923	0	0	32.39	100	20	7	91.1
**565**	852	0	216	0	0	0	10.8	177	923	0	0	32.36	100	20	7	101.9
**566**	863	0	216	0	0	0	0	177	923	0	156	32.37	100	20	7	103.7
**567**	647	216	216	0	0	0	0	177	923	0	156	32.37	100	20	7	118.1
**568**	647	0	216	0	0	216	0	177	923	0	156	32.37	100	20	7	118.7
**569**	432	216	216	0	0	216	0	177	923	0	156	32.40	100	20	7	117.6
**570**	829	0	216	0	0	0	34.5	177	923	0	156	32.39	100	20	7	118.7
**571**	852	0	216	0	0	0	10.8	177	923	0	156	32.36	100	20	7	122.6
**572**	863	0	216	0	0	0	0	177	923	0	0	32.37	100	20	28	104.1
**573**	647	216	216	0	0	0	0	177	923	0	0	32.37	100	20	28	109
**574**	647	0	216	0	0	216	0	177	923	0	0	32.37	100	20	28	109
**575**	432	216	216	0	0	216	0	177	923	0	0	32.40	100	20	28	103
**576**	829	0	216	0	0	0	34.5	177	923	0	0	32.39	100	20	28	113.9
**577**	852	0	216	0	0	0	10.8	177	923	0	0	32.36	100	20	28	113.3
**578**	863	0	216	0	0	0	0	177	923	0	156	32.37	100	20	28	142.1
**579**	647	216	216	0	0	0	0	177	923	0	156	32.37	100	20	28	157.2
**580**	647	0	216	0	0	216	0	177	923	0	156	32.37	100	20	28	156
**581**	432	216	216	0	0	216	0	177	923	0	156	32.40	100	20	28	154.4
**582**	829	0	216	0	0	0	34.5	177	923	0	156	32.39	100	20	28	156
**583**	852	0	216	0	0	0	10.8	177	923	0	156	32.36	100	20	28	154.4
**584**	900	0	220	0	0	0	0	163	1005	0	156	40.00	100	23	7	124.1
**585**	900	0	165	0	0	55	0	163	1021	0	156	40.00	100	23	7	115.5
**586**	900	0	110	0	0	110	0	163	1036	0	156	40.00	100	23	7	113.5
**587**	810	0	220	0	0	90	0	163	1002	0	156	40.00	100	23	7	116
**588**	720	0	220	0	0	180	0	163	998	0	156	40.00	100	23	7	116.5
**589**	630	0	220	0	0	270	0	163	994	0	156	40.00	100	23	7	110
**590**	900	0	220	0	0	0	0	163	1005	0	156	40.00	100	23	14	132.9
**591**	900	0	165	0	0	55	0	163	1021	0	156	40.00	100	23	14	123
**592**	900	0	110	0	0	110	0	163	1036	0	156	40.00	100	23	14	122
**593**	810	0	220	0	0	90	0	163	1002	0	156	40.00	100	23	14	125.3
**594**	720	0	220	0	0	180	0	163	998	0	156	40.00	100	23	14	125.1
**595**	630	0	220	0	0	270	0	163	994	0	156	40.00	100	23	14	124.5
**596**	900	0	220	0	0	0	0	163	1005	0	156	40.00	100	23	28	143
**597**	900	0	165	0	0	55	0	163	1021	0	156	40.00	100	23	28	138.8

**598**	900	0	110	0	0	110	0	163	1036	0	156	40.00	100	23	28	138.2
**599**	810	0	220	0	0	90	0	163	1002	0	156	40.00	100	23	28	140.4
**600**	720	0	220	0	0	180	0	163	998	0	156	40.00	100	23	28	132.2
**601**	630	0	220	0	0	270	0	163	994	0	156	40.00	100	23	28	130.6
**602**	900	0	220	0	0	0	0	163	1005	0	156	40.00	100	23	56	161.6
**603**	900	0	165	0	0	55	0	163	1021	0	156	40.00	100	23	56	154.8
**604**	900	0	110	0	0	110	0	163	1036	0	156	40.00	100	23	56	152.4
**605**	810	0	220	0	0	90	0	163	1002	0	156	40.00	100	23	56	157.2
**606**	720	0	220	0	0	180	0	163	998	0	156	40.00	100	23	56	153.7
**607**	630	0	220	0	0	270	0	163	994	0	156	40.00	100	23	56	151
**615**	666.4	35.1	210.5	0	259.6	0	0	164.2	820.8	0	156	17.50	100	90	28	144.7
**616**	631.4	70.1	210.5	0	259.6	0	0	164.2	820.8	0	156	17.50	100	90	28	159.1
**617**	596.3	105.2	210.5	0	259.6	0	0	164.2	820.8	0	156	17.50	100	90	28	147.4
**618**	561.2	140.3	210.5	0	259.6	0	0	164.2	820.8	0	156	17.50	100	90	28	139.6
**619**	526.1	175.4	210.5	0	259.6	0	0	164.2	820.8	0	156	17.50	100	90	28	130.6
**620**	701.5	0	210.5	0	259.6	0	0	164.2	820.8	0	156	17.50	100	200	28	161.9
**621**	666.4	35.1	210.5	0	259.6	0	0	164.2	820.8	0	156	17.50	100	200	28	170.1
**622**	631.4	70.1	210.5	0	259.6	0	0	164.2	820.8	0	156	17.50	100	200	28	175.9
**624**	561.2	140.3	210.5	0	259.6	0	0	164.2	820.8	0	156	17.50	100	200	28	179.1
**626**	620.2	0	114.2	0	0	164.9	0	161.9	1277.5	0	156	36.00	100	20	1	91.1
**627**	558.1	62	114.2	0	0	164.9	0	161.9	1276.6	0	156	36.00	100	20	1	77.9
**630**	620.2	0	114.2	0	0	164.9	0	161.9	1277.5	0	156	36.00	100	20	3	113.8
**631**	558.1	62	114.2	0	0	164.9	0	161.9	1276.6	0	156	36.00	100	20	3	102.7
**634**	620.2	0	114.2	0	0	164.9	0	161.9	1277.5	0	156	36.00	100	20	7	134.2
**635**	558.1	62	114.2	0	0	164.9	0	161.9	1276.6	0	156	36.00	100	20	7	130.5
**636**	496.1	124	114.2	0	0	164.9	0	161.9	1275.9	0	156	36.00	100	20	7	129.8
**637**	434.1	186	114.2	0	0	164.9	0	161.9	1275	0	156	36.00	100	20	7	121.7
**639**	558.1	62	114.2	0	0	164.9	0	161.9	1276.6	0	156	36.00	100	20	14	160.5
**640**	496.1	124	114.2	0	0	164.9	0	161.9	1275.9	0	156	36.00	100	20	14	162
**641**	434.1	186	114.2	0	0	164.9	0	161.9	1275	0	156	36.00	100	20	14	153.9
**643**	558.1	62	114.2	0	0	164.9	0	161.9	1276.6	0	156	36.00	100	20	28	170.7
**644**	496.1	124	114.2	0	0	164.9	0	161.9	1275.9	0	156	36.00	100	20	28	175.8
**645**	434.1	186	114.2	0	0	164.9	0	161.9	1275	0	156	36.00	100	20	28	168.4
**646**	863.2	0	215.8	0	0	0	0	177	1079	0	0	21.60	100	20	1	36.1
**647**	857.8	0	215.8	0	0	0	5.4	177	1079	0	0	21.60	100	20	1	42.2
**648**	852.4	0	215.8	0	0	0	10.8	177	1079	0	0	21.60	100	20	1	47.7
**649**	847	0	215.8	0	0	0	16.2	177	1079	0	0	21.60	100	20	1	53.1
**650**	841.6	0	215.8	0	0	0	21.6	177	1079	0	0	21.60	100	20	1	53.1
**651**	863.2	0	215.8	0	0	0	0	177	1079	0	0	21.60	100	20	3	75.6
**652**	857.8	0	215.8	0	0	0	5.4	177	1079	0	0	21.60	100	20	3	81.7
**653**	852.4	0	215.8	0	0	0	10.8	177	1079	0	0	21.60	100	20	3	85.1
**654**	847	0	215.8	0	0	0	16.2	177	1079	0	0	21.60	100	20	3	87.8
**655**	841.6	0	215.8	0	0	0	21.6	177	1079	0	0	21.60	100	20	3	81.7
**656**	863.2	0	215.8	0	0	0	0	177	1079	0	0	21.60	100	20	7	83.7
**657**	857.8	0	215.8	0	0	0	5.4	177	1079	0	0	21.60	100	20	7	92.6
**658**	852.4	0	215.8	0	0	0	10.8	177	1079	0	0	21.60	100	20	7	102.1
**659**	847	0	215.8	0	0	0	16.2	177	1079	0	0	21.60	100	20	7	105.5
**660**	841.6	0	215.8	0	0	0	21.6	177	1079	0	0	21.60	100	20	7	102.8
**661**	863.2	0	215.8	0	0	0	0	177	1079	0	0	21.60	100	20	28	103.5
**662**	857.8	0	215.8	0	0	0	5.4	177	1079	0	0	21.60	100	20	28	108.3
**663**	852.4	0	215.8	0	0	0	10.8	177	1079	0	0	21.60	100	20	28	113.7
**664**	847	0	215.8	0	0	0	16.2	177	1079	0	0	21.60	100	20	28	112.3
**665**	841.6	0	215.8	0	0	0	21.6	177	1079	0	0	21.60	100	20	28	110.3
**666**	863.2	0	215.8	0	0	0	0	177	1079	0	0	21.60	100	20	90	119.8
**667**	857.8	0	215.8	0	0	0	5.4	177	1079	0	0	21.60	100	20	90	128
**668**	852.4	0	215.8	0	0	0	10.8	177	1079	0	0	21.60	100	20	90	130.7
**669**	847	0	215.8	0	0	0	16.2	177	1079	0	0	21.60	100	20	90	123.2
**670**	841.6	0	215.8	0	0	0	21.6	177	1079	0	0	21.60	100	20	90	117.8
**671**	750	0	144	0	0	200	0	182	990	0	0	38.00	100	20	3	82.3
**672**	675	75	144	0	0	200	0	182	990	0	0	38.00	100	20	3	68.9
**673**	600	150	144	0	0	200	0	182	990	0	0	36.00	100	20	3	64.5
**674**	525	225	144	0	0	200	0	182	990	0	0	36.00	100	20	3	60.7
**675**	450	300	144	0	0	200	0	182	990	0	0	34.00	100	20	3	53

**676**	375	375	144	0	0	200	0	182	990	0	0	30.00	100	20	3	48.1
**677**	750	0	144	0	0	200	0	182	990	0	0	38.00	100	20	7	100.1
**678**	675	75	144	0	0	200	0	182	990	0	0	38.00	100	20	7	90
**679**	600	150	144	0	0	200	0	182	990	0	0	36.00	100	20	7	84.3
**680**	525	225	144	0	0	200	0	182	990	0	0	36.00	100	20	7	82.3
**681**	450	300	144	0	0	200	0	182	990	0	0	34.00	100	20	7	78
**682**	375	375	144	0	0	200	0	182	990	0	0	30.00	100	20	7	70.3
**683**	750	0	144	0	0	200	0	182	990	0	0	38.00	100	20	28	121.8
**684**	675	75	144	0	0	200	0	182	990	0	0	38.00	100	20	28	125.2
**685**	600	150	144	0	0	200	0	182	990	0	0	36.00	100	20	28	127.6
**686**	525	225	144	0	0	200	0	182	990	0	0	36.00	100	20	28	127.1
**687**	450	300	144	0	0	200	0	182	990	0	0	34.00	100	20	28	122.3
**688**	375	375	144	0	0	200	0	182	990	0	0	30.00	100	20	28	116
**689**	960	0	240	0	0	0	0	234	793.7	0	0	45.00	100	23	7	107.6
**690**	960	0	240	0	0	0	0	234	787.1	0	19.5	45.00	100	23	7	119.3
**691**	960	0	240	0	0	0	0	234	780.5	0	39	45.00	100	23	7	121.8
**692**	960	0	240	0	0	0	0	234	773.8	0	58.5	45.00	100	23	7	124.2
**693**	960	0	240	0	0	0	0	234	767.2	0	78	45.00	100	23	7	126.9
**694**	960	0	240	0	0	0	0	234	754	0	117	45.00	100	23	7	129.2
**695**	960	0	240	0	0	0	0	234	740.7	0	156	45.00	100	23	7	136.6
**696**	960	0	240	0	0	0	0	234	786.4	0	19.5	45.00	100	23	7	118.1
**697**	960	0	240	0	0	0	0	234	779.2	0	39	45.00	100	23	7	120.2
**698**	960	0	240	0	0	0	0	234	771.9	0	58.5	45.00	100	23	7	122.6
**699**	960	0	240	0	0	0	0	234	764.7	0	78	45.00	100	23	7	127.4
**700**	960	0	240	0	0	0	0	234	720.7	0	117	57.00	100	23	7	130
**701**	960	0	240	0	0	0	0	234	706.2	0	156	57.00	100	23	7	132.4
**702**	960	0	240	0	0	0	0	234	793.7	0	0	45.00	100	23	28	136.6
**703**	960	0	240	0	0	0	0	234	787.1	0	19.5	45.00	100	23	28	148.7
**704**	960	0	240	0	0	0	0	234	780.5	0	39	45.00	100	23	28	150.1
**705**	960	0	240	0	0	0	0	234	773.8	0	58.5	45.00	100	23	28	152.5
**706**	960	0	240	0	0	0	0	234	767.2	0	78	45.00	100	23	28	154.8
**707**	960	0	240	0	0	0	0	234	754	0	117	45.00	100	23	28	160.6
**708**	960	0	240	0	0	0	0	234	740.7	0	156	45.00	100	23	28	162.8
**709**	960	0	240	0	0	0	0	234	786.4	0	19.5	45.00	100	23	28	144.5
**710**	960	0	240	0	0	0	0	234	779.2	0	39	45.00	100	23	28	148.6
**711**	960	0	240	0	0	0	0	234	771.9	0	58.5	45.00	100	23	28	151.8
**712**	960	0	240	0	0	0	0	234	764.7	0	78	45.00	100	23	28	154.8
**713**	960	0	240	0	0	0	0	234	720.7	0	117	57.00	100	23	28	156.2
**714**	960	0	240	0	0	0	0	234	706.2	0	156	57.00	100	23	28	160.1
**715**	960	0	240	0	0	0	0	234	793.7	0	0	45.00	100	23	56	150.1
**716**	960	0	240	0	0	0	0	234	787.1	0	19.5	45.00	100	23	56	163.1
**717**	960	0	240	0	0	0	0	234	780.5	0	39	45.00	100	23	56	166.2
**718**	960	0	240	0	0	0	0	234	773.8	0	58.5	45.00	100	23	56	167.6
**719**	960	0	240	0	0	0	0	234	767.2	0	78	45.00	100	23	56	170.7
**720**	960	0	240	0	0	0	0	234	754	0	117	45.00	100	23	56	172.7
**721**	960	0	240	0	0	0	0	234	740.7	0	156	45.00	100	23	56	173.2
**722**	960	0	240	0	0	0	0	234	786.4	0	19.5	45.00	100	23	56	160.9
**723**	960	0	240	0	0	0	0	234	779.2	0	39	45.00	100	23	56	163.3
**724**	960	0	240	0	0	0	0	234	771.9	0	58.5	45.00	100	23	56	164.7
**725**	960	0	240	0	0	0	0	234	764.7	0	78	45.00	100	23	56	164.9
**726**	960	0	240	0	0	0	0	234	720.7	0	117	57.00	100	23	56	165.5
**727**	960	0	240	0	0	0	0	234	706.2	0	156	57.00	100	23	56	166.7
**728**	960	0	240	0	0	0	0	234	793.7	0	0	45.00	100	23	90	156.1
**729**	960	0	240	0	0	0	0	234	787.1	0	19.5	45.00	100	23	90	167.8
**730**	960	0	240	0	0	0	0	234	780.5	0	39	45.00	100	23	90	169.4
**731**	960	0	240	0	0	0	0	234	773.8	0	58.5	45.00	100	23	90	170.5
**732**	960	0	240	0	0	0	0	234	767.2	0	78	45.00	100	23	90	173.6
**733**	960	0	240	0	0	0	0	234	754	0	117	45.00	100	23	90	176.8
**734**	960	0	240	0	0	0	0	234	740.7	0	156	45.00	100	23	90	180.3
**735**	960	0	240	0	0	0	0	234	786.4	0	19.5	45.00	100	23	90	165.1
**736**	960	0	240	0	0	0	0	234	779.2	0	39	45.00	100	23	90	167.7
**737**	960	0	240	0	0	0	0	234	771.9	0	58.5	45.00	100	23	90	167.9
**738**	960	0	240	0	0	0	0	234	764.7	0	78	45.00	100	23	90	169.1

**739**	960	0	240	0	0	0	0	234	720.7	0	117	57.00	100	23	90	170.9
**740**	960	0	240	0	0	0	0	234	706.2	0	156	57.00	100	23	90	172.5
**741**	960	0	240	0	0	0	0	234	793.7	0	0	45.00	100	23	180	161.3
**742**	960	0	240	0	0	0	0	234	787.1	0	19.5	45.00	100	23	180	169.8
**743**	960	0	240	0	0	0	0	234	780.5	0	39	45.00	100	23	180	170.9
**744**	960	0	240	0	0	0	0	234	773.8	0	58.5	45.00	100	23	180	172.3
**745**	960	0	240	0	0	0	0	234	767.2	0	78	45.00	100	23	180	175.2
**746**	960	0	240	0	0	0	0	234	754	0	117	45.00	100	23	180	178.1
**747**	960	0	240	0	0	0	0	234	740.7	0	156	45.00	100	23	180	182.4
**748**	960	0	240	0	0	0	0	234	786.4	0	19.5	45.00	100	23	180	167.9
**749**	960	0	240	0	0	0	0	234	779.2	0	39	45.00	100	23	180	169.1
**750**	960	0	240	0	0	0	0	234	771.9	0	58.5	45.00	100	23	180	170.3
**751**	960	0	240	0	0	0	0	234	764.7	0	78	45.00	100	23	180	172.5
**752**	960	0	240	0	0	0	0	234	720.7	0	117	57.00	100	23	180	174.2
**753**	960	0	240	0	0	0	0	234	706.2	0	156	57.00	100	23	180	177.2
**754**	472	315	262	0	0	0	0	178	1049	0	0	21.00	100	23	7	94.5
**755**	472	315	262	0	0	0	0	178	1049	0	156	21.00	100	23	7	115
**756**	472	315	262	0	0	0	0	178	1049	0	156	21.00	100	23	7	120.9
**757**	472	315	262	0	0	0	0	178	1049	0	156	21.00	100	23	7	108.8
**760**	472	315	262	0	0	0	0	178	1049	0	0	21.00	100	23	28	98.3
**761**	472	315	262	0	0	0	0	178	1049	0	156	21.00	100	23	28	134.3
**762**	472	315	262	0	0	0	0	178	1049	0	156	21.00	100	23	28	143.5
**763**	472	315	262	0	0	0	0	178	1049	0	156	21.00	100	23	28	127.6
**764**	472	315	262	0	0	0	0	178	1049	0	156	21.00	100	23	28	123.4
**765**	472	315	262	0	0	0	0	178	1049	0	156	21.00	100	23	28	120.5
**766**	680	0	120	0	0	0	0	160	1462	0	0	9.59	100	23	7	63.7
**767**	640	0	160	0	0	0	0	160	1446.4	0	0	9.60	100	23	7	57.4
**768**	600	0	200	0	0	0	0	160	1434	0	0	9.60	100	23	7	60.5
**769**	680	0	120	0	0	0	0	144	1502.8	0	0	9.59	100	23	7	68.49
**770**	680	0	120	0	0	0	0	176	1421.2	0	0	9.59	100	23	7	55.32
**771**	680	0	120	0	0	0	0	160	1462	0	0	11.22	100	23	7	63.22
**772**	680	0	120	0	0	0	0	160	1468.8	0	0	8.02	100	23	7	58.83
**773**	765	0	135	0	0	0	0	180	1315.8	0	0	10.79	100	23	7	66.8
**774**	720	0	180	0	0	0	0	180	1303.2	0	0	10.80	100	23	7	69.2
**775**	675	0	225	0	0	0	0	180	1289.25	0	0	10.80	100	23	7	64.5
**776**	720	0	180	0	0	0	0	162	1353.6	0	0	10.80	100	23	7	72
**777**	720	0	180	0	0	0	0	198	1260	0	0	10.80	100	23	7	64.1
**778**	720	0	180	0	0	0	0	180	1296	0	0	12.60	100	23	7	70.24
**779**	720	0	180	0	0	0	0	180	1310.4	0	0	9.00	100	23	7	67.61
**780**	850	0	150	0	0	0	0	200	1173	0	0	11.99	100	23	7	72.3
**781**	800	0	200	0	0	0	0	200	1160	0	0	12.00	100	23	7	73.1
**782**	750	0	250	0	0	0	0	200	1140	0	0	12.00	100	23	7	67.6
**783**	850	0	150	0	0	0	0	180	1224	0	0	11.99	100	23	7	74.63
**784**	850	0	150	0	0	0	0	220	1122	0	0	11.99	100	23	7	67.61
**785**	850	0	150	0	0	0	0	200	1173	0	0	14.03	100	23	7	72.88
**786**	850	0	150	0	0	0	0	200	1181.5	0	0	10.03	100	23	7	69.37
**787**	680	0	120	0	0	0	0	160	1462	0	0	9.59	100	23	28	118.7
**788**	640	0	160	0	0	0	0	160	1446.4	0	0	9.60	100	23	28	113.2
**790**	680	0	120	0	0	0	0	144	1502.8	0	0	9.59	100	23	28	122.93
**791**	680	0	120	0	0	0	0	176	1421.2	0	0	9.59	100	23	28	108.88
**792**	680	0	120	0	0	0	0	160	1462	0	0	11.22	100	23	28	122.05
**793**	680	0	120	0	0	0	0	160	1468.8	0	0	8.02	100	23	28	111.51
**794**	765	0	135	0	0	0	0	180	1315.8	0	0	10.79	100	23	28	122.6
**795**	720	0	180	0	0	0	0	180	1303.2	0	0	10.80	100	23	28	124.2
**796**	675	0	225	0	0	0	0	180	1289.25	0	0	10.80	100	23	28	123.4
**797**	720	0	180	0	0	0	0	162	1353.6	0	0	10.80	100	23	28	128.2
**798**	720	0	180	0	0	0	0	198	1260	0	0	10.80	100	23	28	122.05
**799**	720	0	180	0	0	0	0	180	1296	0	0	12.60	100	23	28	127.32
**800**	720	0	180	0	0	0	0	180	1310.4	0	0	9.00	100	23	28	125.56
**801**	850	0	150	0	0	0	0	200	1173	0	0	11.99	100	23	28	128.1
**802**	800	0	200	0	0	0	0	200	1160	0	0	12.00	100	23	28	126.6
**803**	750	0	250	0	0	0	0	200	1140	0	0	12.00	100	23	28	125
**804**	850	0	150	0	0	0	0	180	1224	0	0	11.99	100	23	28	132.59

**805**	850	0	150	0	0	0	0	220	1122	0	0	11.99	100	23	28	122.93
**806**	850	0	150	0	0	0	0	200	1173	0	0	14.03	100	23	28	129.95
**807**	850	0	150	0	0	0	0	200	1181.5	0	0	10.03	100	23	28	127.32
**808**	729	0	182	0	0	0	0	182	920	230	156	27.00	100	20	28	148
**809**	365	365	183	0	0	0	0	183	920	230	156	17.00	100	20	28	151
**810**	480	0	171	0	0	206	0	171	920	230	156	33.00	100	20	28	157
**813**	480	0	171	0	0	206	0	171	920	230	156	33.00	100	210	28	172
**814**	863	0	216	0	0	0	0	177	1079	0	0	21.60	100	23	1	35.68
**815**	858	0	216	0	0	0	5.4	177	1079	0	0	21.60	100	23	1	42.39
**816**	852	0	216	0	0	0	10.8	177	1079	0	0	21.60	100	23	1	48.2
**817**	847	0	216	0	0	0	16.2	177	1079	0	0	21.60	100	23	1	53.38
**818**	842	0	216	0	0	0	21.6	177	1079	0	0	21.60	100	23	1	53.08
**819**	863	0	216	0	0	0	0	177	1079	0	0	21.60	100	23	3	75.05
**820**	858	0	216	0	0	0	5.4	177	1079	0	0	21.60	100	23	3	82.07
**821**	852	0	216	0	0	0	10.8	177	1079	0	0	21.60	100	23	3	85.12
**822**	847	0	216	0	0	0	16.2	177	1079	0	0	21.60	100	23	3	87.88
**823**	842	0	216	0	0	0	21.6	177	1079	0	0	21.60	100	23	3	82.09
**824**	863	0	216	0	0	0	0	177	1079	0	0	21.60	100	23	7	92.48
**825**	858	0	216	0	0	0	5.4	177	1079	0	0	21.60	100	23	7	92.79
**826**	852	0	216	0	0	0	10.8	177	1079	0	0	21.60	100	23	7	101.64
**827**	847	0	216	0	0	0	16.2	177	1079	0	0	21.60	100	23	7	106.22
**828**	842	0	216	0	0	0	21.6	177	1079	0	0	21.60	100	23	7	103.18
**829**	863	0	216	0	0	0	0	177	1079	0	0	21.60	100	23	28	103.81
**830**	858	0	216	0	0	0	5.4	177	1079	0	0	21.60	100	23	28	108.09
**831**	852	0	216	0	0	0	10.8	177	1079	0	0	21.60	100	23	28	113.58
**832**	847	0	216	0	0	0	16.2	177	1079	0	0	21.60	100	23	28	112.68
**833**	842	0	216	0	0	0	21.6	177	1079	0	0	21.60	100	23	28	110.25
**834**	863	0	216	0	0	0	0	177	1079	0	0	21.60	100	23	28	119.41
**835**	858	0	216	0	0	0	5.4	177	1079	0	0	21.60	100	23	28	128.26
**836**	852	0	216	0	0	0	10.8	177	1079	0	0	21.60	100	23	28	129.79
**837**	847	0	216	0	0	0	16.2	177	1079	0	0	21.60	100	23	28	124
**838**	842	0	216	0	0	0	21.6	177	1079	0	0	21.60	100	23	28	117.62
**839**	1251.2	0	291.3	0	0	0	0	201.4	407.8	0	0	28.20	100	23	1	82.81
**840**	825.8	0	192.3	486.2	0	0	0	201.4	444.4	0	0	10.50	100	23	1	70.14
**841**	575.6	0	134	772.2	0	0	0	201.4	455.7	0	0	5.10	100	23	1	33.49
**842**	325.3	0	75.7	1058.2	0	0	0	201.4	455.4	0	0	5.20	100	23	1	28.51
**843**	1251.2	0	291.3	0	0	0	0	201.4	407.8	0	0	28.20	100	23	3	112.692
**844**	825.8	0	192.3	486.2	0	0	0	201.4	444.4	0	0	10.50	100	23	3	116.31
**846**	325.3	0	75.7	1058.2	0	0	0	201.4	455.4	0	0	5.20	100	23	3	54.32
**847**	1251.2	0	291.3	0	0	0	0	201.4	407.8	0	0	28.20	100	23	7	124.94
**850**	325.3	0	75.7	1058.2	0	0	0	201.4	455.4	0	0	5.20	100	23	7	75.62
**851**	1251.2	0	291.3	0	0	0	0	201.4	407.8	0	0	28.20	100	23	14	140.83
**852**	825.8	0	192.3	486.2	0	0	0	201.4	444.4	0	0	10.50	100	23	14	148.52
**853**	575.6	0	134	772.2	0	0	0	201.4	455.7	0	0	5.10	100	23	14	142.64
**854**	325.3	0	75.7	1058.2	0	0	0	201.4	455.4	0	0	5.20	100	23	14	97.84
**855**	1251.2	0	291.3	0	0	0	0	201.4	407.8	0	0	28.20	100	23	28	147.27
**856**	825.8	0	192.3	486.2	0	0	0	201.4	444.4	0	0	10.50	100	23	28	167.18
**857**	575.6	0	134	772.2	0	0	0	201.4	455.7	0	0	5.10	100	23	28	155.87
**858**	325.3	0	75.7	1058.2	0	0	0	201.4	455.4	0	0	5.20	100	23	28	113.34
**859**	1251.2	0	291.3	0	0	0	0	201.4	407.8	0	0	28.20	100	23	56	156.53
**861**	575.6	0	134	772.2	0	0	0	201.4	455.7	0	0	5.10	100	23	56	171
**862**	325.3	0	75.7	1058.2	0	0	0	201.4	455.4	0	0	5.20	100	23	56	123.05
**863**	788.5	0	433.7	0	0	0	0	160.3	867.4	0	0	52.60	100	23	28	195.2
**864**	788.5	0	433.7	0	0	0	0	160.3	867.4	0	156	52.60	100	23	28	220.2
**865**	788.5	0	433.7	0	0	0	0	160.3	867.4	0	156	52.60	100	23	28	209.6
**866**	788.5	0	433.7	0	0	0	0	160.3	867.4	0	156	52.60	100	23	28	201.4
**867**	788.5	0	433.7	0	0	0	0	160.3	867.4	0	156	52.60	100	23	28	218.4
**869**	788.5	0	433.7	0	0	0	0	160.3	867.4	0	156	52.60	100	23	28	218.5
**870**	788.5	0	433.7	0	0	0	0	160.3	867.4	0	156	52.60	100	23	28	205.1
**871**	788.5	0	433.7	0	0	0	0	160.3	867.4	0	39	52.60	100	23	28	209.4
**872**	788.5	0	433.7	0	0	0	0	160.3	867.4	0	78	52.60	100	23	28	199.6
**873**	788.5	0	433.7	0	0	0	0	160.3	867.4	0	117	52.60	100	23	28	209.7
**874**	788.5	0	433.7	0	0	0	0	160.3	867.4	0	39	52.60	100	23	28	198.6

**875**	788.5	0	433.7	0	0	0	0	160.3	867.4	0	78	52.60	100	23	28	208.4
**876**	788.5	0	433.7	0	0	0	0	160.3	867.4	0	117	52.60	100	23	28	220.5
**877**	788.5	0	433.7	0	0	0	0	160.3	867.4	0	39	52.60	100	23	28	211.2
**878**	788.5	0	433.7	0	0	0	0	160.3	867.4	0	78	52.60	100	23	28	186.7
**879**	788.5	0	433.7	0	0	0	0	160.3	867.4	0	117	52.60	100	23	28	218.1
**880**	946	0	71	47	0	166	0	166	344	516	0	35.76	98	23	3	120.77
**881**	784	0	157	235	0	0	0	171	330	493	0	39.66	98	23	3	102.73
**882**	937	0	70	47	0	164	0	164	340	511	78	36.82	98	23	3	108.74
**883**	777	0	155	233	0	0	0	169	326	490	78	40.64	98	23	3	101.09
**884**	946	0	71	47	0	166	0	166	344	516	0	35.76	98	23	14	131.69
**885**	784	0	157	235	0	0	0	171	330	493	0	39.66	98	23	14	118.03
**886**	937	0	70	47	0	164	0	164	340	511	78	36.82	98	23	14	123.5
**887**	777	0	155	233	0	0	0	169	326	490	78	40.64	98	23	14	110.93
**888**	946	0	71	47	0	166	0	166	344	516	0	35.76	98	23	28	144.81
**889**	784	0	157	235	0	0	0	171	330	493	0	39.66	98	23	28	127.87
**890**	937	0	70	47	0	164	0	164	340	511	78	36.82	98	23	28	150.82
**891**	777	0	155	233	0	0	0	169	326	490	78	40.64	98	23	28	134.43
**892**	946	0	71	47	0	166	0	166	344	516	0	35.76	98	23	90	160.66
**893**	784	0	157	235	0	0	0	171	330	493	0	39.66	98	23	90	150.82
**894**	937	0	70	47	0	164	0	164	340	511	78	36.82	98	23	90	166.67
**895**	777	0	155	233	0	0	0	169	326	490	78	40.64	98	23	90	151.91
**898**	792	0	264	0	0	0	0	173	1056	0	156	21.10	100	20	3	102.86
**899**	784	0	261	0	0	0	0	171	1045	0	234	20.90	100	20	3	106
**900**	809	0	270	0	0	0	0	177	1079	0	0	21.60	100	20	7	92.77
**901**	800	0	267	0	0	0	0	175	1067	0	78	21.30	100	20	7	110.42
**902**	792	0	264	0	0	0	0	173	1056	0	156	21.10	100	20	7	123.66
**903**	784	0	261	0	0	0	0	171	1045	0	234	20.90	100	20	7	128.07
**904**	809	0	270	0	0	0	0	177	1079	0	0	21.60	100	20	28	106
**905**	800	0	267	0	0	0	0	175	1067	0	78	21.30	100	20	28	129.96
**906**	792	0	264	0	0	0	0	173	1056	0	156	21.10	100	20	28	150.13
**907**	784	0	261	0	0	0	0	171	1045	0	234	20.90	100	20	28	158.32
**909**	800	0	267	0	0	0	0	175	1067	0	78	21.30	100	20	90	148.87
**910**	792	0	264	0	0	0	0	173	1056	0	156	21.10	100	20	90	161.47
**911**	784	0	261	0	0	0	0	171	1045	0	234	20.90	100	20	90	166.51

© 2020 by the authors. Submitted for possible open access publication under the terms and conditions of the Creative Commons Attribution (CC BY) license (http://creativecommons.org/licenses/by/4.0/).
